# LRRK2: Molecular Mechanisms in Parkinson’s Disease

**DOI:** 10.3390/ijms27177606

**Published:** 2026-08-25

**Authors:** Oscar Arias-Carrión, Magdalena Guerra-Crespo, Daniel Ortuño-Sahagún, Emmanuel Ortega-Robles

**Affiliations:** 1 División de Neurociencias, Clínica, Instituto Nacional de Rehabilitación Luis Guillermo Ibarra Ibarra, Mexico City 14389, Mexico; 2 Escuela de Medicina y Ciencias de la Salud, Tecnologico de Monterrey, Mexico City 14380, Mexico; 3 Laboratory of Regenerative Medicine, Department of Physiology, Faculty of Medicine, National Autonomous University of Mexico, Mexico City 04510, Mexico; mguerra@facmed.unam.mx; 4 Laboratorio de Neuroinmunobiología Molecular, Instituto de Neurociencias Traslacionales, CUCS, Universidad de Guadalajara, Guadalajara 44350, Mexico; daniel.ortuno.sahagun@gmail.com

**Keywords:** LRRK2, Parkinson’s disease, Rab GTPases, cryo–electron microscopy, lysosomal stress, conformational regulation

## Abstract

Leucine-rich repeat kinase 2 (LRRK2) has emerged as a central molecular node linking genetic risk, membrane trafficking, lysosomal homeostasis, and immune signalling in Parkinson’s disease (PD). Rather than functioning as a conventional protein kinase, LRRK2 operates as a conformationally regulated, Rab-directed signalling machine whose activity is governed by long-range interdomain communication, membrane recruitment, and cooperative interactions with small GTPases. Converging advances in cryo-electron microscopy, quantitative phosphoproteomics, and human genetics indicate that pathogenic mutations, lysosomal stress, and pharmacological inhibitors do not simply alter catalytic output, but reshape the conformational landscape of LRRK2, biasing it toward distinct structural states with divergent cellular consequences. A defining feature of this system is the selective phosphorylation of Rab GTPases at low stoichiometry—most prominently Rab8 and Rab10—yet with disproportionate functional impact on vesicle trafficking, ciliogenesis, autophagy, and organelle positioning. The identification of Rab-directed phosphatases, particularly PPM1H, further establishes that LRRK2 signalling is governed by a dynamically balanced kinase–phosphatase circuit operating in space and time. These observations, together with emerging evidence linking LRRK2 activation to lysosomal damage and immune pathways, support a unifying hypothesis: PD-associated LRRK2 dysfunction arises from maladaptive stabilization of specific conformational and spatial states within a membrane-responsive signalling network, leading to persistent misregulation of Rab-dependent trafficking and organelle homeostasis, rather than from kinase hyperactivity alone. In this review, we integrate structural, biochemical, and cellular evidence to advance this framework and discuss its implications for disease mechanisms and therapy. We highlight key unresolved challenges—including conformation-selective drug targeting, spatial control of Rab phosphorylation, and context-dependent immune–neuronal crosstalk—and propose that restoring physiological regulation of LRRK2, rather than simply inhibiting its activity, will be essential for achieving mechanism-based disease modification in Parkinson’s disease.

## 1. Introduction

Leucine-rich repeat kinase 2 (LRRK2) occupies a distinctive position at the intersection of structural biology, intracellular signalling, and neurodegeneration. Since the independent identification of pathogenic *LRRK2* mutations as a cause of autosomal dominant Parkinson’s disease (PD) in 2004 [1,2], *LRRK2* has emerged as the most frequent monogenic contributor to PD across diverse populations and as one of the most intensively investigated kinases in human disease biology [3]. Unlike many genes linked to familial neurodegeneration, *LRRK2*-associated PD is clinically indistinguishable from idiopathic disease, exhibits incomplete and age-dependent penetrance, and displays marked neuropathological heterogeneity [4]. These features argue against a simple mutation–phenotype relationship and instead position LRRK2 as a molecular entry point into pathogenic pathways that extend well beyond rare familial cases.

Accumulating genetic, cellular, and biochemical evidence indicates that LRRK2 converges on a set of core processes—vesicular trafficking, lysosomal homeostasis, primary ciliogenesis, and innate immune signalling—that are increasingly recognized as central to PD pathophysiology more broadly [3,5,6]. In this respect, LRRK2 is best viewed not as an isolated genetic outlier but as a regulator of conserved cellular stress-response pathways whose dysregulation may contribute to neuronal vulnerability in a substantial fraction of patients currently classified as having idiopathic PD.

LRRK2 is widely expressed across neural and peripheral tissues, although its abundance varies substantially among organs and cell types. Comparative expression studies have demonstrated prominent LRRK2 expression in peripheral tissues including the lung, kidney, and spleen, whereas within the central nervous system its distribution is regionally heterogeneous [7,8]. In the brain, LRRK2 is broadly expressed, with relatively high levels in the cortex and striatum and lower expression in the substantia nigra in rodent studies [8,9]. LRRK2 is also prominently expressed in peripheral immune populations, including monocytes and B and T lymphocytes, where its abundance can be modulated by inflammatory and microbial stimuli [10]. This broad distribution underscores that LRRK2 biology extends beyond neurons and is likely shaped by tissue- and cell-type-specific physiological contexts.

Over the past decade, advances in cryo-electron microscopy, quantitative phosphoproteomics, and integrative structural modelling have fundamentally reshaped our understanding of LRRK2 function. Rather than behaving as a conventional serine/threonine kinase governed primarily by activation-loop phosphorylation, LRRK2 operates as a conformationally regulated, membrane-responsive signalling hub. High-resolution structural studies reveal that LRRK2 adopts multiple interconverting conformations in which long-range interdomain communication, oligomeric state, and membrane engagement collectively determine kinase output [11,12,13]. These findings are complemented by functional studies demonstrating that phosphorylation of Rab GTPases represents the dominant physiological output of LRRK2 kinase activity in cells and tissues [14,15]. Together, these observations establish LRRK2 as a Rab-directed kinase whose activity is tightly coupled to subcellular localization and membrane context.

In parallel with these mechanistic advances, translational efforts have propelled LRRK2 into the forefront of structure-guided drug discovery for neurodegenerative disease. Multiple ATP-competitive LRRK2 inhibitors have advanced into clinical testing, making LRRK2 one of the few kinases genetically linked to neurodegeneration for which targeted therapies have reached patients. However, this progress has also exposed conceptual and practical tensions. Type I inhibitors stabilize a closed, active-like kinase conformation and induce dephosphorylation of canonical biomarker sites, effects that are accompanied by lung and kidney alterations in preclinical models [16]. By contrast, type II inhibitors that stabilize an inactive kinase conformation suppress Rab phosphorylation without inducing biomarker dephosphorylation but currently lack sufficient selectivity and pharmacokinetic properties for clinical use [12,13]. These observations raise fundamental questions about whether therapeutic efficacy and safety are determined solely by suppression of catalytic activity or, more subtly, by the conformational state in which LRRK2 is trapped.

A further layer of complexity arises from the regulation of Rab phosphorylation itself. Despite robust kinase activation by pathogenic mutations, only a small fraction of total Rab protein is phosphorylated at steady state, yet these low-stoichiometry events exert outsized biological effects [14]. This implies that LRRK2 signalling is governed by spatial precision and local amplification rather than bulk modification. The identification of the Rab-directed phosphatase PPM1H established that Rab phosphorylation is dynamically opposed by specialized phosphatase activity [17], but how kinase–phosphatase balance is orchestrated across distinct membrane compartments, cell types, and physiological states remains poorly understood.

More recently, lysosomal stress and membrane damage have emerged as potent upstream activators of LRRK2, reframing the kinase as both a sensor and effector of endolysosomal integrity [18,19]. This insight links LRRK2 signalling directly to pathways implicated by multiple PD risk genes, including *GBA1*, *ATP13A2*, and *TMEM175*, and suggests that LRRK2 activation may represent a broader cellular response to organelle stress rather than a neuron-specific signalling event [5,20]. Consistent with this broad tissue distribution, LRRK2 activity is particularly relevant in myeloid cells, and genetic associations with inflammatory disorders highlight an intimate connection between LRRK2, immunity, and tissue-specific disease vulnerability [6].

In this review, we synthesize recent structural, biochemical, and cellular insights into LRRK2 biology with an emphasis on unresolved mechanisms and translational implications. We focus on four interrelated challenges: how LRRK2 conformational states are coupled to membrane recruitment and Rab substrate engagement; how kinase–phosphatase balance shapes Rab signalling in space and time; how lysosomal stress, infection, and immune context activate LRRK2 in ways relevant to PD and inflammatory disease; and how evolutionary divergence among *LRRK* paralogues informs both experimental models and therapeutic strategies. By integrating structural principles with cell biology and human genetics, we aim to provide a coherent framework for understanding LRRK2 as a dynamic molecular machine and to clarify how this knowledge may be leveraged to move beyond symptomatic treatment toward mechanism-based disease modification in Parkinson’s disease.

## 2. LRRK2 Structure and Its Conformational Dynamics

High-resolution structural work has reshaped how the field thinks about LRRK2: not as a static kinase, but as a conformationally gated signalling enzyme in which long-range allostery emerges from domain architecture. Early near-atomic cryo–electron microscopy (cryo-EM) reconstructions of the catalytic C-terminal half (ROC–COR–kinase–WD40; “RCKW”) revealed a characteristic J-shaped fold, with WD40, kinase and COR-B aligned along a central axis and the ROC–COR-A unit curving back toward the kinase, immediately suggesting a physical route for GTPase–kinase crosstalk [11].

Subsequent cryo-EM structures of full-length human LRRK2 refined this picture by revealing extensive contacts between the ANK–LRR region and the enzymatic core in the open, autoinhibited conformation, whereas the ARM domain extends away from the core and remains comparatively flexible [21]. In these states, the leucine-rich repeat (LRR) region is positioned in a manner consistent with steric restriction of kinase accessibility. In contrast, the armadillo (ARM) repeats remain comparatively mobile—an arrangement that plausibly couples localization and partner binding to catalytic output [21].

Inhibitor-bound structures have provided a particularly clean readout of the activation landscape. Structures of wild-type and PD-linked variants bound to the type I inhibitor MLi-2 capture the kinase in a closed, active-like conformation. In contrast, the type II inhibitor GZD-824 stabilizes an open, inactive DYG-out state—directly visualizing how inhibitor class can select (rather than merely “block”) specific conformations of the LRRK2 kinase domain [12]. This conformational selectivity matters operationally: it creates a structural rationale for why different inhibitor chemotypes can produce divergent cellular phenotypes despite similar effects on catalytic activity in vitro [12,22].

Beyond the active site, multiple studies converge on discrete structural elements that stabilize the multidomain enzyme and coordinate interdomain coupling. Integrative modelling and dynamics-based analyses highlight helical “bridges” that cap and tether the kinase N-lobe and link ROC–COR to the kinase, providing a mechanistic framework for how interdomain contacts restrain basal activity while permitting rapid switching in response to appropriate cues [23].

At the kinase-domain level, LRRK2 follows the canonical bilobal serine/threonine kinase architecture. Available inactive-state structures consistently show misalignment of regulatory features required for efficient catalysis, providing a straightforward structural explanation for low basal activity in the autoinhibited ensemble [12,21]. Disease-associated substitutions that elevate activity can be understood, in structural terms, as mutations that bias the conformational equilibrium toward closed, active-like states. Consistent with this logic, large-scale functional profiling of PD-linked variants shows that many activating variants reduce phosphorylation at the commonly used N-terminal “biomarker” sites (including Ser935/Ser955/Ser973), underscoring that these sites report a regulated signalling state rather than simple intrinsic kinase output [24].

Microtubule association offers a vivid example of how conformation and oligomeric state translate into cell biology. In situ cryo–electron tomography revealed that the I2020T mutant can assemble as a right-handed double helix around microtubules [25], and subsequent cryo-EM work defined how microtubule-bound assemblies are built from repeating units and stabilized through interfaces involving the ROC surface and higher-order packing [26]. More recently, cryo-electron tomography of full-length LRRK2 further supported the concept that inactive/autoinhibited conformations can nonetheless oligomerize on microtubules under reconstituted conditions [27]. Nevertheless, the physiological meaning of these striking filaments remains unsettled: microtubule association varies across mutants and experimental contexts, and robust microtubule binding by endogenous LRRK2 under physiological conditions has been challenging to establish, cautioning against treating filament formation as a universal proximate driver of pathology [25,26,27].

A complementary regulatory layer comes from membrane recruitment and Rab-dependent assembly. A cryo-EM structure of a Rab29-dependent complex revealed an unexpected tetrameric arrangement in which two central protomers adopt an active-like state. In contrast, peripheral protomers remain inactive, providing a structural mechanism for spatial control and suggesting that higher-order assemblies may be integral to physiological activation at membranes [13]. This reinforces an emerging view: LRRK2 activity is an ensemble property shaped by recruitment, quaternary structure and conformational selection—features that can be exploited pharmacologically, but only if interventions are designed to modulate the conformational landscape without destabilizing the protein [6,13,22].

**Figure 1:**
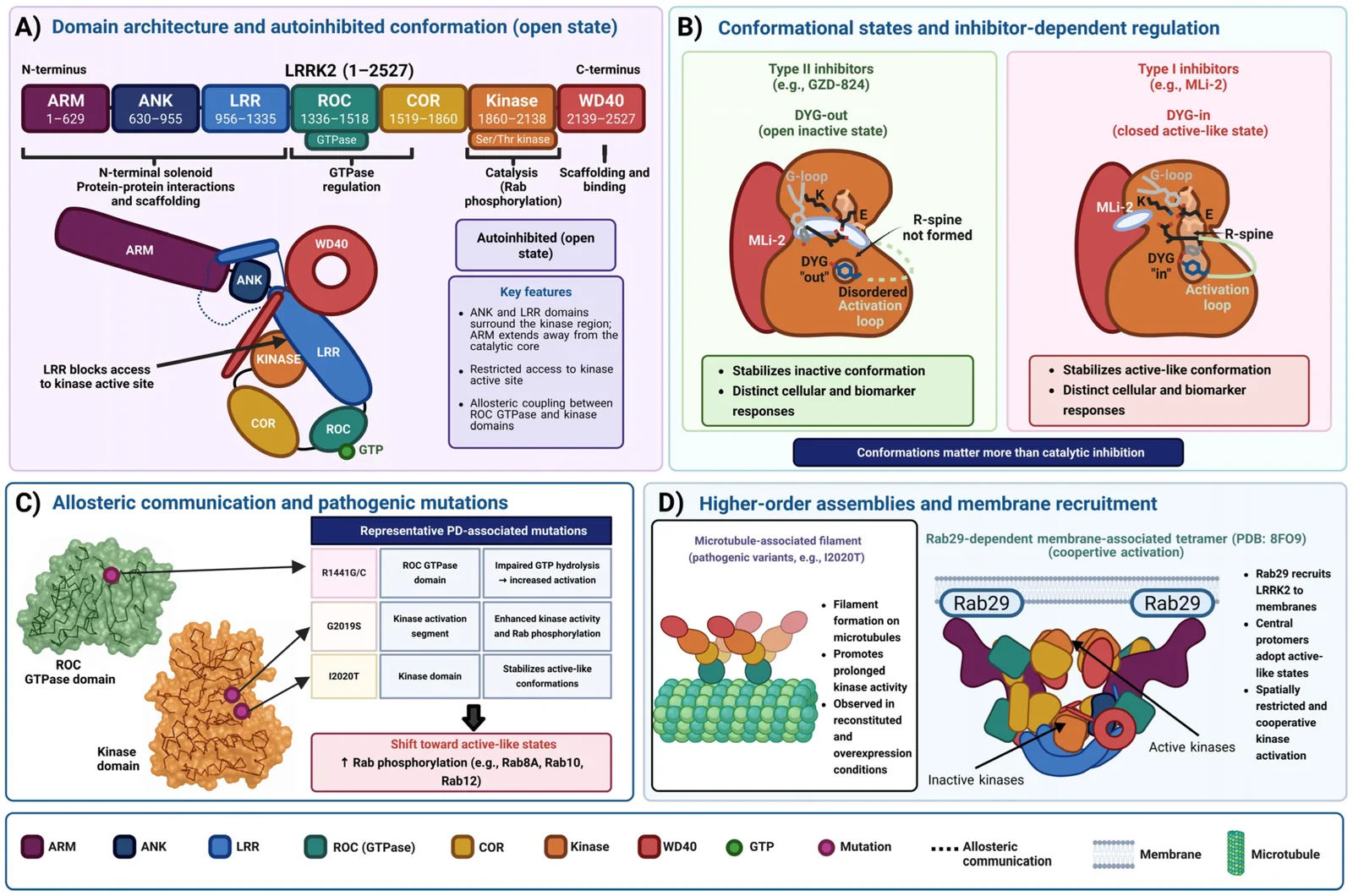
Domain organization, conformational regulation, pathogenic mutations, and higher-order assemblies of LRRK2. (**A**) Domain architecture and autoinhibited open-state model of full-length human LRRK2 (PDB: 7LHW; [21]). The protein consists of the armadillo (ARM), ankyrin (ANK), leucine-rich repeat (LRR), ROC GTPase (ROC), COR, kinase, and WD40 domains. In the experimentally determined full-length structure, the ANK and LRR domains are positioned around the kinase region, whereas the ARM domain extends away from the catalytic core. The LRR domain restricts access to the kinase active site and contributes to the autoinhibited conformation. The arrangement highlights the structural coupling between the ROC GTPase and kinase domains that underlies allosteric regulation. (**B**) Conformational states of the LRRK2 kinase domain and inhibitor-dependent regulation. Type II inhibitors, exemplified by GZD-824 (PDB: 8TZE; [12]), stabilize an open inactive DYG-out conformation characterized by a disordered activation loop, whereas type I inhibitors such as MLi-2 (PDB: 8TXZ; [12]) stabilize a closed active-like DYG-in conformation. These structurally distinct states differentially modulate cellular signalling and biomarker responses despite inhibition of kinase activity. (**C**) Allosteric communication within the catalytic core and representative Parkinson’s disease-associated mutations. Pathogenic substitutions including R1441G/C in the ROC GTPase domain, G2019S in the kinase activation segment, and I2020T in the kinase domain alter interdomain communication, impair GTPase regulation or stabilize active-like conformations, collectively shifting the conformational equilibrium toward enhanced kinase activity and increased Rab phosphorylation (e.g., Rab8A, Rab10, and Rab12). (**D**) Higher-order assemblies and membrane recruitment mechanisms of LRRK2. Left, model of microtubule-associated filament formation (PDB: 6XR4; [25]) observed for selected pathogenic variants, particularly I2020T, which promotes prolonged kinase activity under reconstituted or overexpression conditions. Right, Rab29-dependent membrane-associated tetrameric assembly (PDB: 8FO9; [13,28]), in which Rab29 recruits LRRK2 to cellular membranes, central protomers adopt active-like conformations, and cooperative intermolecular interactions facilitate spatially restricted kinase activation. Created in BioRender. Ledesma Aparicio, J. (2026) https://BioRender.com/fv5o9qm.

Collectively, structural biology has repositioned LRRK2 as a finely tuned molecular machine whose pathogenic states arise from biased equilibria across multiple conformations and assemblies. For translational neuroscience, the implication is concrete: mutations, inhibitors and regulatory partners act primarily by reshaping the structural landscape. Mapping—and ultimately controlling—these states is therefore not descriptive adornment; it is the mechanistic foundation for interpreting biomarker behaviour and for engineering the next generation of LRRK2-targeted therapeutics. The structural organization and conformational regulation of LRRK2 are summarized in Table 1 and illustrated schematically in Figure 1.

## 3. LRRK2: Kinase Signalling and Pathogenic Networks in Parkinson’s Disease

Genetic variation in *LRRK2* represents the most common identifiable cause of inherited PD and provides a uniquely direct molecular entry point into kinase-dependent pathogenic mechanisms that extend well beyond rare monogenic forms of the disorder [1,2]. Building on the structural framework described above, LRRK2 functions as a signalling hub that coordinates GTPase activity, kinase output, and protein–protein interactions involved in intracellular trafficking, cytoskeletal dynamics, and membrane-associated signalling [6].

Pathogenic mutations are non-randomly distributed across the catalytic core, yielding strong genotype–function relationships. The globally prevalent G2019S substitution lies within the activation segment of the kinase domain and reproducibly increases kinase activity [29,30], whereas substitutions affecting Arg1441 within the ROC domain impair GTP hydrolysis and indirectly enhance kinase signalling [14,31]. Despite their distinct biochemical origins, these mutations converge on a shared molecular outcome: a shift toward a hyperactive or dysregulated kinase state rather than simple loss of function, characterized most consistently by increased phosphorylation of endogenous Rab substrates [14,15]. At the cellular level, aberrant LRRK2 activity drives excessive phosphorylation of a defined subset of Rab GTPases, notably Rab8, Rab10 and Rab12 [14,15], leading to perturbations in vesicular trafficking, endolysosomal maturation, autophagic flux and synaptic vesicle recycling [18,32,33,34]—processes now widely recognized as central to dopaminergic-neuron vulnerability.

Although most PD cases remain clinically classified as idiopathic, converging genetic evidence indicates that approximately 10–15% of cases can be attributed to pathogenic variants in a finite group of roughly 20 genes, with *LRRK2* representing the most frequent contributor across diverse populations [3,4]. Large contemporary cohort studies, including multiethnic analyses, estimate that ~3% of individuals with PD carry pathogenic or likely pathogenic *LRRK2* variants. Notably, allele frequencies vary substantially by ancestry, reaching particularly high levels in Ashkenazi Jewish and North African Arab populations, underscoring the importance of population-specific genetic architecture in PD risk stratification.

Clinically, *LRRK2*-associated PD closely resembles idiopathic disease with respect to age at onset and core motor manifestations, rendering recognition challenging in routine practice. Nonetheless, subtle and reproducible distinctions are emerging. Longitudinal cohort studies suggest a comparatively slower rate of motor progression and, in some cohorts, a reduced burden of cognitive decline among *LRRK2* mutation carriers, consistent with partially divergent disease trajectories that may reflect differential engagement of downstream pathogenic pathways [35,36]. Neuropathological findings further emphasize this heterogeneity: *LRRK2*-associated PD may present with classical Lewy body pathology, minimal α-synuclein deposition, or mixed patterns, indicating that LRRK2 dysfunction is not obligatorily coupled to a single proteinopathy.

A defining characteristic of *LRRK2*-related PD is its incomplete and highly variable penetrance, typically estimated between 30% and 70% depending on mutation type, age, ancestry and modifying factors that remain incompletely defined [37,38]. Increasing evidence implicates polygenic risk burden, lysosomal and mitochondrial resilience pathways, inflammatory exposures and environmental factors as modifiers of disease expression. These observations reinforce the view that *LRRK2* mutations operate within a broader genetic and biological landscape rather than dictating deterministic outcomes, posing challenges for genetic counselling and undermining simplistic monogenic interpretations of PD risk.

From a translational perspective, these insights necessitate a conceptual shift in how LRRK2 is framed in both diagnosis and therapy. Rather than treating *LRRK2* mutations as rare genetic curiosities, they are more appropriately understood as entry points into kinase-driven pathogenic networks that converge on vesicular trafficking, lysosomal homeostasis, cytoskeletal regulation and neuroimmune signalling—processes now central to contemporary models of PD pathogenesis [6]. With PD affecting nearly 10 million individuals worldwide and prevalence rising with population ageing, LRRK2 offers a uniquely actionable bridge between molecular mechanism and clinical intervention. Integrating genetic stratification, pathway-informed biomarkers and precisely targeted kinase-modulating therapies holds realistic promise for disease modification, with implications extending beyond mutation carriers to a substantial fraction of patients currently labelled as having idiopathic PD. The principal genetic, molecular, clinical and translational features of *LRRK2*-associated PD are summarized in Table 2.

## 4. LRRK2 as a Conformationally Regulated Kinase in Parkinson’s Disease

LRRK2 is a large, multidomain protein of approximately 288 kDa that integrates scaffolding, GTPase, and serine/threonine kinase activities within a single polypeptide [11,21]. This unusual domain composition places LRRK2 at the intersection of signal transduction, membrane trafficking, and cytoskeletal regulation, enabling coordination of multiple intracellular processes essential for neuronal homeostasis [6]. Rather than functioning as a linear signalling enzyme, LRRK2 operates as a conformationally regulated signalling hub whose activity emerges from dynamic interdomain communication and structural rearrangements [11,21,23].

Building on the structural organization described in Section 2, LRRK2 activity is governed by long-range coupling between its GTPase and kinase modules and by conformational rearrangements that regulate catalytic accessibility and signalling output [11,21,23].

This conformational coupling provides a structural explanation for a defining feature of *LRRK2*-associated Parkinson’s disease: pathogenic variants distributed across multiple domains converge on a shared biochemical phenotype, namely enhanced kinase activity [24]. The most prevalent disease-causing substitution, G2019S, resides within the activation segment of the kinase domain and alters a glycine within the conserved Asp–Tyr–Gly (DYG) motif. Accounting for approximately 80% of pathogenic *LRRK2* variants worldwide, this substitution reproducibly increases kinase activity by roughly twofold in biochemical and cellular assays [29,30]. Importantly, this moderate degree of activation is sufficient to perturb downstream signalling without grossly destabilizing the protein.

Even more pronounced kinase activation is observed with mutations located outside the catalytic site. Variants affecting the ROC domain, including R1441G, R1441C and R1441H, impair GTP hydrolysis and stabilize a GTP-bound state that favours kinase activation. Similarly, the COR-B mutation Y1699C destabilizes the ROC–COR interface, indirectly enhancing kinase output. When quantified using phosphorylation of endogenous Rab GTPase substrates—most notably Rab10—these mutations often produce three- to fourfold increases in kinase activity [14,15]. These findings underscore that LRRK2 dysregulation arises not solely from perturbation of the active site, but from disruption of allosteric control mechanisms that normally restrain kinase function.

Large-scale sequencing efforts have identified more than 780 missense variants in *LRRK2*, the majority of which are likely benign. To distinguish pathogenic from neutral variation, a functional classification framework has emerged that quantitatively compares mutant kinase activity with wild-type LRRK2 activity in cellular systems. An increase of approximately 1.5-fold or greater is generally considered indicative of pathogenic activation. Using this approach, roughly 200–220 variants detected in individuals with PD have been systematically evaluated, of which around 50 demonstrate clear kinase-activating properties. Strikingly, many of these variants map outside the kinase domain, including substitutions within the ARM, ANK and LRR regions, as well as within the ROC and COR-B domains. This distribution reinforces the view of LRRK2 as an allosterically regulated enzyme whose activity reflects long-range structural communication rather than the state of a single catalytic pocket [11,21,23,24].

Population-specific variants further illustrate the continuum between benign polymorphisms and pathogenic mutations. The COR-A substitution R1628P, which occurs at relatively high frequency in East Asian populations, confers only modest kinase activation. Meta-analyses indicate that this variant functions as a weak genetic risk factor rather than a fully penetrant mutation, highlighting how subtle conformational biases can influence disease susceptibility without deterministically causing PD [47,48]. Additional substitutions within the kinase domain itself, such as I2020T and T2031S, demonstrate that multiple structural routes—ranging from activation segment destabilization to altered αC-helix positioning—can bias LRRK2 toward an active conformation.

Post-translational modifications provide an additional, clinically informative layer of regulation of LRRK2. Endogenous LRRK2 is constitutively phosphorylated at several serine residues located in a disordered region proximal to the LRR domain, most prominently Ser910 and Ser935, together with Ser955 and Ser973. These sites are phosphorylated at high stoichiometry, largely by casein kinase 1α (*CSNK1A1*), despite their atypical sequence context. Phosphorylation at these residues promotes binding to 14-3-3 adaptor proteins, stabilizing LRRK2 in an inactive, cytosolic state. Loss of phosphorylation at these so-called “biomarker sites” accompanies kinase activation and membrane association, making them widely used surrogate markers of LRRK2 pathway activity and practical readouts of target engagement in preclinical and clinical trials of kinase inhibitors [6,14,49,50,51].

By contrast, Ser1292 represents an autophosphorylation site that more directly reports intrinsic kinase activity. Pathogenic mutations, particularly G2019S, robustly enhance Ser1292 phosphorylation in overexpression systems [52,53]. However, detection of endogenous Ser1292 phosphorylation remains technically challenging owing to low stoichiometry, limited antibody sensitivity and unfavourable mass spectrometric properties of the corresponding peptide. This limitation highlights a persistent translational gap between mechanistic insight and biomarker feasibility, underscoring the need for improved analytical strategies to quantify LRRK2 activity in patient-derived material.

Collectively, the structural, molecular and genetic features of LRRK2 redefine it as a dynamically regulated signalling platform rather than a simple kinase. Pathogenicity arises not from isolated mutations, but from failure of conformational control that biases the protein toward sustained kinase hyperactivity. This framework provides a unifying explanation for the phenotypic convergence of diverse variants and carries direct therapeutic implications: effective intervention will require pathway-level modulation rather than mutation-specific correction. In this respect, LRRK2 serves as both a mechanistic anchor and a translational paradigm for kinase-driven neurodegeneration in Parkinson’s disease. The molecular mechanisms linking LRRK2 conformational regulation, pathogenic variation and kinase activation are summarized in Table 3.

## 5. LRRK2 Phosphorylates Rab GTPases

For more than a decade, identification of the physiological substrates of LRRK2 remained elusive. This difficulty stemmed from the low endogenous expression of LRRK2, its modest basal kinase activity, and the limited specificity of early pharmacological inhibitors. A decisive breakthrough came with the work of Steger and colleagues, who combined quantitative phosphoproteomics with genetic and chemical orthogonality to define bona fide in vivo substrates of LRRK2 [14]. By analysing phosphoproteomic changes in mouse embryonic fibroblasts expressing the hyperactive LRRK2 (G2019S) variant before and after treatment with two structurally distinct LRRK2 inhibitors, they identified a small set of phosphosites consistently suppressed by kinase inhibition. To rigorously control for off-target effects, the authors engineered knock-in cells expressing the LRRK2 (A2016T) inhibitor-resistant variant. In this setting, phosphosites reduced by MLi-2 in wild-type but not A2016T-expressing cells could be confidently attributed to LRRK2 activity. Remarkably, the intersection of these datasets yielded only two shared phosphopeptides: LRRK2 phosphorylated at Ser935 and phosphorylated Rab10. Subsequent work established Ser1292 as a genuine autophosphorylation site, but the identification of Rab GTPases as primary substrates provided a unifying mechanistic framework for LRRK2 signalling in both physiology and disease [14,15].

The relevance of Rab phosphorylation to PD is underscored by the observation that all established pathogenic *LRRK2* mutations enhance Rab phosphorylation in cells and tissues, an effect consistently reversed by structurally diverse classes of LRRK2 inhibitors [14,15,29]. Although additional candidate substrates have been proposed, including ribosomal protein S15, these findings have not yet met the same level of genetic and pharmacological validation. Current evidence therefore supports Rab GTPases as the dominant physiological targets of LRRK2.

Rab proteins comprise a large family of small Ras-like GTPases that define membrane identity and orchestrate vesicular trafficking throughout secretory and endocytic pathways. By cycling between inactive GDP-bound and active GTP-bound states under the control of guanine nucleotide exchange factors and GTPase-activating proteins, Rab proteins coordinate vesicle budding, transport along cytoskeletal tracks, and docking and fusion at target membranes [61]. Of the approximately 65 Rab proteins encoded in the human genome, LRRK2 selectively phosphorylates a restricted subset, including Rab3 isoforms, Rab8A/B, Rab10, Rab12, Rab29, Rab35 and Rab43 [14]. In specific genetic contexts, such as cells carrying the PD-associated *VPS35* (D620N) retromer mutation, LRRK2 activity is further amplified, leading to robust phosphorylation of additional Rab species, notably Rab1 [62]. By contrast, evidence supporting Rab5 as a physiologically relevant endogenous substrate remains limited and context-dependent.

In experimental and translational studies, LRRK2 pathway activity is most reliably monitored using phospho-specific antibodies against Rab substrates or by mass spectrometry–based quantification of phospho-Rab peptides in cells, animal models and human biospecimens. In contrast, detection of endogenous LRRK2 autophosphorylation at Ser1292 remains technically challenging, even in knock-in models harbouring activating mutations, owing to its low stoichiometry and analytical limitations [44].

At the molecular level, Rab proteins are anchored to membranes by C-terminal geranylgeranyl modifications and are dynamically extracted and recycled by GDP dissociation inhibitors (GDIs). Their interactions with effector proteins depend on conformational changes in two flexible regions—Switch I and Switch II—that report the nucleotide state. LRRK2 phosphorylates Rab substrates at conserved residues within the Switch II region, such as Thr73 in Rab10, Thr72 in Rab8A and Ser106 in Rab12. This modification disrupts binding to GDIs, impairs interactions with most canonical effectors, and, in the case of Rab8A, blocks engagement with its guanine nucleotide exchange factor Rabin8. Consequently, phosphorylated Rabs are predicted to become trapped on membranes at sites of modification, unable to undergo normal cycles of extraction and reactivation [14,39,61].

Notably, the biological impact of this pathway does not require widespread Rab modification. Quantitative analyses indicate that even under conditions of LRRK2 hyperactivation, less than 1% of a given Rab pool is phosphorylated at steady state. Nevertheless, phospho-Rabs acquire the ability to bind a distinct set of effector proteins, including RILPL1, RILPL2, JIP3, JIP4 and myosin Va, which recognize the phosphorylated Switch II motif via a conserved RILP homology 2 (RH2) domain. These high-affinity interactions are sufficient to elicit dominant cellular phenotypes, whereas extensive Rab phosphorylation would likely be incompatible with cell viability owing to global disruption of membrane trafficking [14,33,34,39].

One of the most compelling downstream consequences of aberrant LRRK2–Rab signalling is inhibition of primary cilium formation. Binding of phospho-Rab10 to RILPL1 suppresses ciliogenesis in cultured cells and in vivo within discrete brain regions, including the dorsal striatum and somatosensory cortex. In the striatum, loss of primary cilia impairs Hedgehog signalling in cholinergic interneurons, reducing their capacity to support dopaminergic neurons via glial cell–derived neurotrophic factor (GDNF). This pathway provides a direct mechanistic link between aberrant Rab phosphorylation and selective neuronal vulnerability in PD [39,63]. Beyond ciliogenesis, elevated LRRK2-driven Rab phosphorylation disrupts axonal autophagosome transport and perturbs centrosomal and centriolar cohesion, implicating this pathway in fundamental aspects of neuronal maintenance and polarity.

Collectively, the identification of Rab GTPases as principal substrates of LRRK2 represents a conceptual inflection point in PD research. It reframes LRRK2 as a master regulator of vesicular trafficking whose pathological activation selectively rewires Rab-dependent pathways. This insight provides a coherent mechanistic bridge between genetic risk, subcellular dysfunction and circuit-level vulnerability, while offering tractable biomarkers and rational therapeutic targets for disease-modifying intervention. The substrate hierarchy, molecular mechanisms and pathogenic consequences of the LRRK2–Rab signalling axis are summarized in Table 4.

## 6. LRRK2 Membrane Recruitment and Activation

Under basal conditions, LRRK2 resides predominantly in the cytosol, with only a minor fraction—approximately 5–10%—associated with intracellular membranes. Early biochemical and cell-based studies nonetheless converged on a central principle: LRRK2 exhibits substantially higher kinase activity when localized to membrane surfaces. It is now well established that this spatial regulation is mediated primarily by Rab GTPases, which recruit LRRK2 to membranes and locally activate it to phosphorylate nearby Rab substrates. Multiple Rab-interaction interfaces within the N-terminal ARM domain of LRRK2 provide the structural basis for this process. Because GTP-bound Rabs are intrinsically membrane-anchored through C-terminal prenylation, their engagement with LRRK2 is sufficient to drive both membrane association and catalytic activation [6,54,67,68].

Initial insight into Rab-dependent activation emerged from studies of Rab29 (also known as *RAB7L1*), encoded within the PARK16 locus, a PD risk region identified by genome-wide association studies. Overexpression experiments demonstrated that Rab29 localizes to the Golgi apparatus and potently recruits and activates LRRK2, leading to robust phosphorylation of Rab substrates. However, genetic loss-of-function studies indicate that Rab29 is unlikely to serve as a universal physiological activator of LRRK2. Across multiple Rab29 knockout cell lines and tissues, basal Rab phosphorylation levels remain largely unchanged, and pharmacological stimuli that recruit LRRK2 to lysosomes—such as nigericin, monensin, chloroquine or L-leucyl-L-leucine methyl ester—still elicit normal LRRK2-dependent Rab phosphorylation in the absence of Rab29. These observations must be interpreted in the context of Rab abundance: Rab29 is expressed at relatively low copy number in most cell types compared with Rab8A and Rab10, which are among the most abundant Rabs in mammalian cells. By contrast, Rab29 is highly expressed in immune cells, where its depletion markedly reduces LRRK2 activation in macrophages, and related Rabs such as Rab38 appear to fulfil analogous roles in monocytes. Together, these findings support a model in which Rab29 and its close homologues function as cell-type–specific regulators of LRRK2, particularly within immune and myeloid lineages, rather than as ubiquitous activators [6,13,54,68,69].

At the molecular level, Rab-mediated recruitment of LRRK2 is orchestrated through multiple, functionally distinct binding sites within the ARM domain. Dephosphorylated, GTP-bound Rab29, together with Rab32 and Rab38, binds to a region encompassing ARM residues ~360–450, commonly referred to as Rab-binding Site 1. This interaction has been visualized directly in cryo–electron microscopy structures of full-length LRRK2 and is supported by AlphaFold-based structural modelling. Importantly, Site 1 is not selective for Rab29: it also binds Rab8A and Rab10 with micromolar affinity, a property likely to be physiologically relevant given the high abundance of these Rabs in most cell types. Mutation of key interface residues, such as Leu403, abolishes binding and disrupts membrane recruitment, confirming the functional importance of this site. Notably, Site 1 recognizes only nonphosphorylated Rab proteins, thereby preferentially engaging substrates prior to LRRK2-mediated modification [13,54,68].

A second Rab-binding interface, Site 2, resides at the extreme N terminus of LRRK2 and displays the opposite specificity: it selectively binds phosphorylated Rab GTPases. Mutation of Lys17 or Lys18 disrupts phospho-Rab binding at this site and markedly reduces LRRK2 colocalization with Rabs and membrane association in cells. Reconstitution experiments using purified LRRK2 and synthetic lipid bilayers demonstrated that membrane recruitment proceeds through a cooperative, feed-forward mechanism. Initial engagement of nonphosphorylated Rabs at Site 1 brings LRRK2 to the membrane, where it phosphorylates local Rab substrates. The newly generated phospho-Rabs then bind to Site 2, increasing avidity and stabilizing LRRK2 at the membrane surface. This dual engagement markedly reduces dissociation, amplifies local kinase concentration and promotes the formation of membrane microdomains enriched in phosphorylated Rabs that efficiently recruit downstream effector proteins [54].

A third Rab-interaction site within the ARM domain, Site 3, was identified through unbiased genome-wide CRISPR screening and Rab-focused RNA interference approaches. This site, located between Sites 1 and 2, selectively binds Rab12 and is required for Rab12-dependent activation of LRRK2. Mutations of residues such as Glu240 or Ser244 disrupt Rab12 binding and attenuate Rab10 phosphorylation in cellular systems. Although Rab12 can activate LRRK2 independently of Site 2 in overexpression models, structural and biochemical data indicate that simultaneous occupancy of multiple Rab-binding sites is feasible and likely contributes to the robustness and context dependence of LRRK2 activation [54,70].

Across all three sites, Rab GTPases bind LRRK2 in their GTP-loaded conformations via residues within their Switch II regions. This binding mode sequesters the very serine or threonine residues that are targets of LRRK2-mediated phosphorylation, indicating that ARM-domain engagement alone cannot position Rab substrates for catalysis. These observations lead to an important conceptual conclusion: in addition to ARM-domain docking sites that mediate membrane recruitment and retention, LRRK2 must harbour a distinct substrate-recognition interface within or proximal to its kinase domain that properly orients Rab proteins for phosphorylation of the Switch II motif. Identification of this catalytic docking surface remains a major open challenge and represents a potential therapeutic target orthogonal to ATP-competitive inhibition [6,54,70].

Collectively, these findings redefine LRRK2 activation as a spatially and temporally regulated process driven by cooperative Rab interactions at membrane surfaces. Rather than acting as a diffusely active cytosolic kinase, LRRK2 functions as a membrane-responsive signalling hub whose activity is amplified through feed-forward Rab phosphorylation. This framework provides a mechanistic link between genetic risk, subcellular localization and pathway activation, and highlights membrane recruitment as a central determinant of LRRK2-driven pathology and a promising target for precision therapeutics in Parkinson’s disease. The mechanisms governing Rab-dependent membrane recruitment, cooperative retention and feed-forward activation of LRRK2 are summarized in Table 5.

## 7. LRRK2 Conformation and Type I Versus Type II Inhibitors

A unifying feature of PD–associated *LRRK2* mutations is their capacity to enhance kinase activity toward Rab substrates, most consistently Rab10, across cellular, animal and human-derived systems. This convergence provides a compelling mechanistic rationale for targeting LRRK2 kinase activity as a disease-modifying strategy in PD [3,14]. Accordingly, pharmacological inhibition of LRRK2 has emerged as one of the most advanced precision-medicine approaches currently under clinical evaluation. To date, all selective LRRK2 inhibitors that have progressed into human studies—including DNL201, DNL151 (BIIB122), and the widely used preclinical compound MLi-2—belong to the class of type I kinase inhibitors [6,72].

Type I inhibitors bind competitively within the ATP-binding pocket of the kinase domain and stabilize LRRK2 in a closed conformation that closely resembles the catalytically active state. A defining pharmacodynamic signature of type I LRRK2 inhibition is robust dephosphorylation of the N-terminal biomarker sites Ser910 and Ser935, which normally support binding of 14-3-3 adaptor proteins and stabilize LRRK2 in the cytosol [49,50]. Notably, many pathogenic *LRRK2* variants—including substitutions in the ROC–COR domains and the kinase-domain mutation I2020T—exhibit a similar loss of biomarker-site phosphorylation even in the absence of inhibitors [49,51]. These parallel effects support a conformational model in which either pathogenic mutations or type I inhibitors bias LRRK2 toward a closed, active-like state that renders these serine residues inaccessible to upstream kinases or more susceptible to dephosphorylation. Although alanine substitution of biomarker residues does not alter intrinsic kinase activity in vitro, it modestly reduces basal Rab phosphorylation in cells, indicating that these sites fine-tune rather than dictate signalling output [14].

Loss of 14-3-3 binding due to dephosphorylation of the biomarker site has important structural and cellular consequences. Several studies report that either type I inhibitor treatment or mutation of biomarker sites promotes LRRK2 destabilization and aggregation, particularly in overexpression systems [49,59]. These observations have gained renewed relevance following cryo–electron microscopy analyses revealing tetrameric assemblies of LRRK2 composed of kinase-active and kinase-inactive subunits. If such assemblies occur under physiological conditions, enforced stabilization of a uniformly closed, active-like conformation by type I inhibitors could perturb native oligomeric equilibria and potentially favour aberrant, aggregation-prone states [12,13].

Consistent with this possibility, both pathogenic *LRRK2* mutations and type I inhibitors promote association of overexpressed LRRK2 with microtubules, resulting in impaired organelle transport and altered motor protein function [25,73]. Importantly, however, there is currently no convincing evidence that endogenous LRRK2—either wild type or mutant—binds microtubules under physiological conditions or following pharmacological inhibition. This discrepancy highlights a critical gap between structural and overexpression-based observations and their relevance to disease biology. Definitive resolution will require strategies that selectively disrupt microtubule-binding interfaces in endogenous LRRK2 without altering kinase activity.

Safety considerations have further sharpened interest in conformation-specific pharmacology. Multiple structurally distinct type I inhibitors induce dose-dependent, largely reversible morphological changes in the lungs and kidneys of rodents and nonhuman primates, characterized by vacuolation and altered lysosomal morphology in type II pneumocytes [16,74]. At high doses, certain highly selective type I inhibitors have been associated with irreversible pneumocyte hyperplasia and fibrotic changes in preclinical studies. By contrast, individuals heterozygous for loss-of-function *LRRK2* variants—who retain one functional allele—do not exhibit overt pulmonary, renal or hepatic phenotypes, despite the relative frequency of such variants in the general population [75]. Whether a complete loss of LRRK2 function is tolerated in humans remains unknown, as homozygous null individuals have not yet been systematically identified. These observations raise a fundamental question: are the toxicities observed with type I inhibitors driven by on-target suppression of kinase activity, by stabilization of a nonphysiological, active-like conformation, by disruption of 14-3-3 binding, or by compound-specific off-target effects?

These uncertainties have renewed interest in type II kinase inhibitors as an alternative therapeutic strategy. Unlike type I compounds, type II inhibitors bind preferentially to the inactive conformation of the kinase, exploiting a pocket that becomes accessible upon activation-loop rearrangement and stabilization of the DYG-out state. Structural analyses of LRRK2 bound to the type II inhibitor GZD-824 reveal partial overlap with the ATP-binding site alongside engagement of a distinct allosteric back pocket, accompanied by pronounced conformational changes in the glycine-rich loop [12,13]. By stabilizing an inactive conformation, type II inhibitors suppress kinase activity without inducing biomarker-site dephosphorylation, loss of 14-3-3 binding or microtubule association in cellular systems [49,60].

Although currently available type II LRRK2 inhibitors lack sufficient selectivity and pharmacokinetic properties for clinical application, proof-of-concept studies demonstrate that this class can effectively suppress Rab phosphorylation while preserving biomarker-site phosphorylation. These features suggest that type II inhibitors may decouple therapeutic efficacy from some of the liabilities associated with type I compounds. Recent high-resolution structures of LRRK2 in complex with GZD-824 therefore provide an important foundation for structure-guided medicinal chemistry aimed at developing selective, brain-penetrant type II inhibitors [6].

From a translational perspective, the distinction between type I and type II LRRK2 inhibitors is not merely pharmacological but conceptual. It reflects a deeper appreciation that LRRK2 functions as a conformationally regulated signalling hub and that therapeutic success may depend as much on stabilizing appropriate structural states as on suppressing catalytic activity per se. Comparative monitoring of downstream biomarkers—notably Rab10 phosphorylation and Ser935 status—will be essential for benchmarking these strategies in preclinical and clinical settings. A central unresolved question remains whether LRRK2 inhibition, by either approach, can meaningfully slow disease progression in idiopathic PD, where LRRK2 activity appears dysregulated even in the absence of pathogenic *LRRK2* mutations. Addressing this question will require long-term, biomarker-informed clinical trials that integrate molecular mechanisms with patient-centred outcomes. The principal structural, pharmacodynamic and translational differences between type I and type II LRRK2 inhibitors are summarized in Table 6.

## 8. PPM1H: A Rab-Specific Phosphatase That Counterbalances LRRK2 Signalling

Phosphorylation of Rab GTPases by LRRK2 is a highly dynamic and tightly regulated process. When cells are acutely exposed to LRRK2 inhibitors, phosphorylation of Rab substrates—most notably Rab10—declines within minutes, whereas dephosphorylation of the canonical LRRK2 biomarker sites Ser910 and Ser935 proceeds on a substantially slower timescale, typically over 40–80 min [14]. This kinetic dissociation indicates that one or more highly active phosphatases directly oppose Rab phosphorylation and that Rab dephosphorylation constitutes an independent, rapidly responsive arm of the LRRK2 pathway rather than a passive consequence of reduced kinase activity.

A systematic effort to identify the phosphatase responsible for Rab dephosphorylation was undertaken by Berndsen and colleagues, who performed a focused RNA interference screen targeting 264 phosphatase catalytic subunits and 56 regulatory components [17]. Using phospho-specific Rab10 antibodies as a functional readout, they identified protein phosphatase magnesium-dependent 1H (PPM1H) as a key negative regulator of LRRK2 signalling. Depletion of PPM1H selectively increased Rab10 phosphorylation without altering total Rab10 or LRRK2 protein levels, providing strong evidence that PPM1H directly counteracts LRRK2-mediated Rab phosphorylation rather than indirectly modulating kinase abundance or activity.

To establish substrate specificity, Berndsen et al. exploited classical principles of phosphatase biology and engineered a substrate-trapping mutant of PPM1H (D288A). This catalytically inactive variant retains substrate-binding capacity but is unable to complete dephosphorylation and release. Immunoprecipitation of PPM1H (D288A), followed by mass spectrometry, revealed selective enrichment of Rab8A, Rab8B and Rab10, with minimal recovery of unrelated proteins. Notably, Rab12 was not captured, underscoring a high degree of substrate selectivity in cells. This specificity is particularly striking given that Rab8A can also be phosphorylated at an independent site (Ser111) by a PINK1-regulated kinase. PPM1H selectively removed the LRRK2-dependent phospho-Thr72 modification while sparing the PINK1-linked site, demonstrating exquisite molecular discrimination between distinct phospho-epitopes on the same substrate [17].

Structural analyses have further clarified the mechanistic basis of this selectivity. High-resolution studies revealed that PPM1H contains an unusual ~110–amino-acid “flap” domain adjacent to its catalytic core, a feature absent from many related PPM family phosphatases [17]. Domain-swapping experiments showed that this flap is both necessary and sufficient to confer phospho-Rab specificity: transplantation of the PPM1H flap into the closely related phosphatase PPM1J endowed the latter with the ability to dephosphorylate LRRK2-modified Rab proteins. These findings establish PPM1H as a specialized phosphatase that has evolved structural adaptations to recognize phosphorylated Rab GTPases with high fidelity.

The apparent lack of Rab12 dephosphorylation by PPM1H in intact cells initially appeared paradoxical, given that Rab12 is a bona fide LRRK2 substrate. Subsequent work indicates that intracellular localization is a key determinant. PPM1H is enriched at the Golgi apparatus and shows greater spatial overlap with Rab8A and Rab10 than with Rab12. Consistent with this interpretation, purified PPM1H efficiently dephosphorylates phospho-Rab12 in vitro, indicating that the enzyme is intrinsically capable of targeting this substrate but may be spatially segregated from it in cells [79]. These observations imply that additional Rab-directed phosphatases operate in parallel with PPM1H to ensure compartment-specific control of Rab phosphorylation across distinct membrane domains. Indeed, residual Rab dephosphorylation persists in PPM1H-deficient cells following LRRK2 inhibition, albeit at a reduced rate, supporting the existence of complementary phosphatase activities.

Beyond its biochemical role, PPM1H has emerged as a powerful experimental lever for dissecting the functional consequences of LRRK2–Rab signalling. Manipulation of PPM1H levels enables selective amplification or suppression of Rab phosphorylation without altering LRRK2 directly. For example, depletion of PPM1H is sufficient to suppress primary cilium formation in otherwise wild-type cells, providing strong evidence that LRRK2 regulates ciliogenesis through Rab phosphorylation rather than through alternative substrates [17]. More recently, similar approaches have demonstrated that LRRK2-dependent regulation of axonal autophagosome transport is mediated through Rab phosphorylation, linking this signalling axis to neuronal maintenance and intracellular trafficking in a disease-relevant context [34].

Collectively, the identification of PPM1H as a Rab-specific phosphatase completes a critical regulatory loop within the LRRK2 signalling pathway. It reveals that pathogenic LRRK2 signalling is defined not only by kinase hyperactivation but also by the balance between phosphorylation and dephosphorylation of Rab substrates. From a translational perspective, this insight reframes LRRK2 signalling as a tuneable circuit rather than a unidirectional cascade. Modulating phosphatase activity, spatial organization, or the kinase–phosphatase balance may therefore be as important as direct kinase inhibition in efforts to normalize Rab-dependent trafficking defects in PD. The kinetic, structural, spatial and functional mechanisms by which PPM1H counter-regulates LRRK2–Rab signalling are summarized in Table 7.

## 9. Lysosomal Damage and Other Activators of LRRK2

Although LRRK2 is predominantly cytosolic under basal conditions, a growing body of evidence indicates that lysosomal stress is a potent and context-dependent trigger of its activation. Early studies demonstrated that exposure to lysosomotropic agents such as chloroquine, which alkalinize lysosomes and induce organelle swelling, promotes recruitment of LRRK2 to lysosomal membranes in a cell-type–specific manner, occurring robustly in macrophages but far less efficiently in fibroblasts or microglial cell lines [18]. This relocalization is accompanied by the appearance of LRRK2 Rab substrates—most notably Rab8A—on lysosomal surfaces, despite Rab8 and Rab10 not being canonical lysosomal Rabs under basal conditions. These observations provided an early indication that lysosomal perturbation can rewire Rab compartmentalization and engage LRRK2 signalling at the endolysosomal interface.

This paradigm was subsequently extended to infectious contexts. Herbst and colleagues showed that intracellular pathogens, including *Mycobacterium*, *Listeria* and *Candida* species, activate LRRK2 in macrophages by inducing phagosomal membrane damage [19]. Such damage promotes the accumulation of phosphorylated Rab8A at phagosomes and coordinates the recruitment of the endosomal sorting complexes required for transport (ESCRT) machinery to facilitate membrane repair. Comparable patterns of LRRK2 recruitment and Rab phosphorylation are observed following treatment with L-leucyl-L-leucine methyl ester (LLOME), which causes lysosomal membrane rupture through intraluminal polymerization and membrane perforation [19,81]. Together, these findings position LRRK2 as a stress-responsive kinase activated by breaches in endolysosomal integrity rather than as a constitutively active cytosolic enzyme.

A recent study identified the STING–CASM–GABARAP pathway as an additional mechanism linking lysosomal perturbation to LRRK2 activation. Activation of stimulator of interferon genes (STING) promotes conjugation of ATG8 proteins to single membranes (CASM), resulting in GABARAP lipidation at lysosomal membranes. GABARAP then interacts with LRRK2 and is required for efficient lysosomal recruitment and kinase activation. Importantly, several chemically induced perturbations of lysosomal homeostasis also converge on this CASM-dependent mechanism, suggesting that it represents a broader pathway coupling lysosomal stress and innate immune signalling to LRRK2 activity [82].

These observations align with a broader reappraisal of lysosomal biology. Lysosomes undergo low-level, spontaneous membrane damage as part of normal cellular physiology and are repaired by rapid, multilayered mechanisms analogous to plasma membrane wound healing. ESCRT components are recruited within seconds to sites of lysosomal disruption to seal small lesions, whereas more extensive damage triggers galectin accumulation and downstream signalling responses [83,84]. In parallel, ESCRT-independent pathways operate through calcium-dependent sphingomyelin scrambling, in which ceramide generated by neutral sphingomyelinase induces inward membrane curvature to promote resealing [85]. Additional repair routes involve phosphatidylinositol 4-phosphate–dependent recruitment of oxysterol-binding protein–related proteins, which establish membrane contact sites between damaged lysosomes and the endoplasmic reticulum (ER), enabling rapid lipid transfer to restore membrane integrity [86,87]. The lipid transfer protein ATG2 further contributes to this process by mediating ER-to-lysosome lipid flux independently of canonical autophagy pathways [88].

Lysosomal dysfunction represents a central and convergent feature of PD, and multiple PD-associated genes—including *LRRK2*, *GBA1*, *ATP13A2* and *TMEM175*—directly regulate lysosomal homeostasis [5,20,89]. Elevated LRRK2 kinase activity impairs lysosomal degradative capacity and autophagic flux in neuronal and immune cells, effects that are reversed by LRRK2 inhibition [90,91,92]. Consistent with this, lysosomal damage—whether induced by infection or chemical stress—drives recruitment of LRRK2 to lysosomal membranes, where it becomes activated and phosphorylates Rab substrates. This process recruits the motor adaptor JIP4, promoting lysosomal tubulation along microtubules and potentially altering lysosome positioning and trafficking [33]. Beyond acute signalling, LRRK2 also exerts longer-term control over lysosomal capacity through transcriptional mechanisms. In macrophages and microglia, active LRRK2 suppresses the abundance and nuclear localization of transcription factor E3 (TFE3), a master regulator of lysosome biogenesis, thereby dampening lysosomal hydrolase expression and reducing proteolytic activity [93,94].

Genetic interactions between *LRRK2* and *GBA1* further underscore the clinical relevance of this axis. Homozygous loss-of-function *GBA1* mutations cause Gaucher disease, whereas heterozygous variants—present in up to 10% of individuals with PD—confer substantial disease risk with incomplete penetrance [95]. Hyperactive LRRK2 signalling has been associated with reduced apparent lysosomal glucocerebrosidase activity and with accumulation of PD-relevant lipids, including bis(monoacylglycerol)phosphates and glycosphingolipids, providing a mechanistic link between LRRK2 activity, lipid dysregulation and lysosomal stress [96,97].

Despite these advances, the mechanisms that recruit LRRK2 to damaged lysosomes remain incompletely resolved. One model proposes that lysosomal stress induces aberrant localization of specific Rab GTPases, which directly recruit LRRK2. In support of this, Rab12 deficiency significantly attenuates—though does not abolish—LRRK2 activation in response to LLOME, indicating partial redundancy among Rab-dependent recruitment pathways [54,65]. An alternative, non-mutually exclusive model posits that lysosomal stress initially stabilizes phosphorylated Rabs via phospho-Rab–binding effectors, which then promote the secondary recruitment of LRRK2. Proteins such as TMEM55B, which anchors JIP4 to lysosomal membranes, and RUFY3, an Arl8 effector, may participate in this process by coupling Rab phosphorylation to dynein-mediated lysosome transport [98,99]. Once phosphorylated, mislocalized Rabs are resistant to extraction by GDP dissociation inhibitors, amplifying their retention at inappropriate compartments and reinforcing LRRK2 recruitment.

An instructive genetic example of this feed-forward model is provided by the PD-associated *VPS35* (D620N) mutation. *VPS35* encodes a core component of the retromer complex that governs retrograde trafficking from endosomes to the Golgi. The D620N mutation causes autosomal dominant PD and enhances LRRK2 kinase activity more robustly than most *LRRK2* mutations themselves [62]. Although the mutation lies outside the canonical cargo-binding site, structural and functional studies suggest that it perturbs retromer oligomerization and endosomal trafficking, leading to lysosomal dysfunction [100]. A parsimonious model proposes that impaired retrograde transport promotes lysosomal stress, triggering LRRK2 recruitment and Rab phosphorylation at lysosomes. Phosphorylated Rabs then recruit the effector RILPL1, which interacts with the lysosomal membrane protein TMEM55B, stabilizing phospho-Rabs on lysosomal membranes and perpetuating aberrant signalling [33].

**Figure 2:**
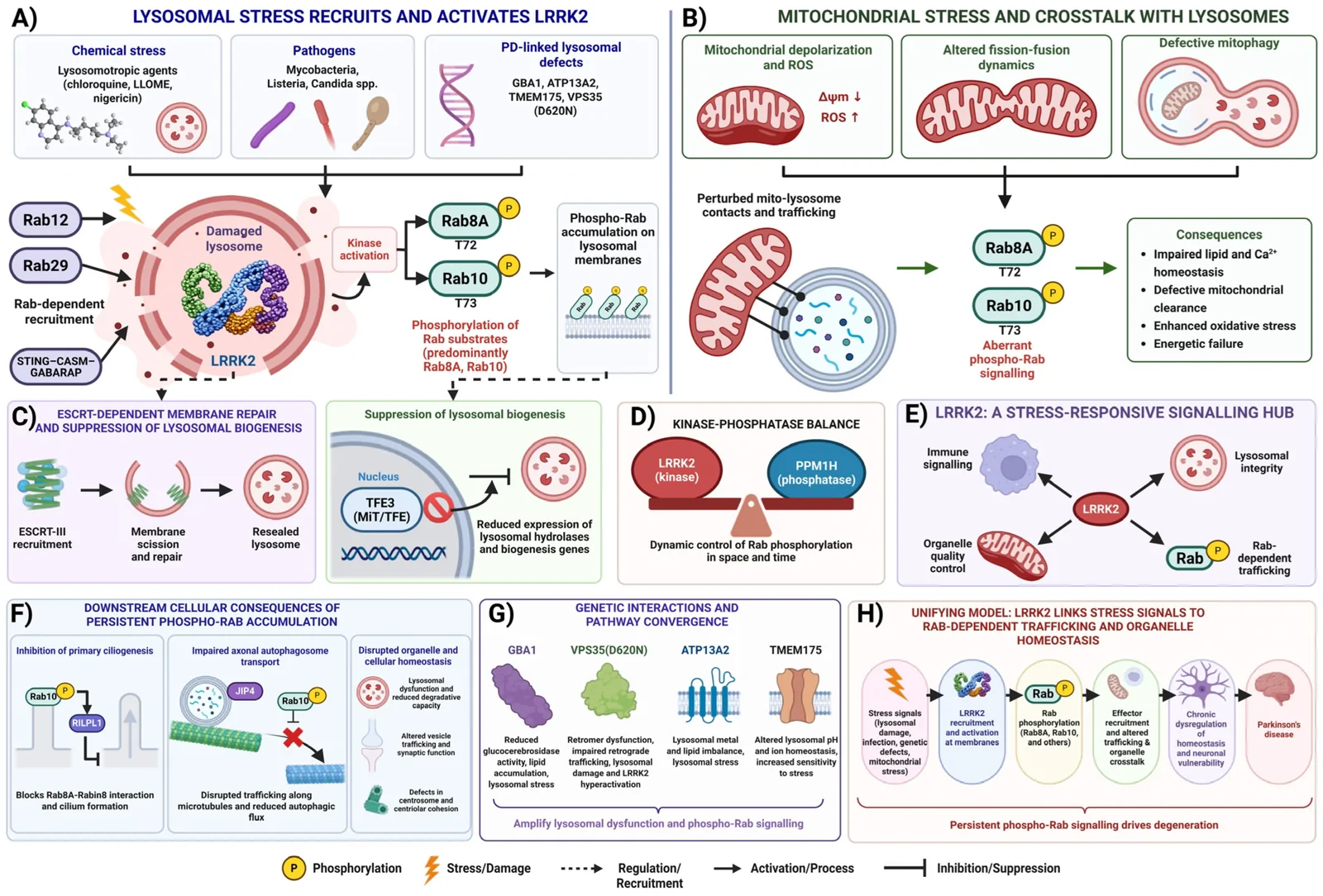
Integrated model of LRRK2-driven stress signalling and phospho-Rab-mediated cellular dysfunction in Parkinson’s disease. (**A**) Lysosomal stress induced by lysosomotropic agents, pathogens, or PD-associated lysosomal defects recruits and activates LRRK2 at damaged lysosomal membranes through Rab-dependent and STING–CASM–GABARAP mechanisms, resulting in phosphorylation of Rab substrates and accumulation of phospho-Rabs on lysosomal membranes. (**B**) Mitochondrial dysfunction, including depolarization, altered fission–fusion dynamics, and defective mitophagy, converges with lysosomal pathways through aberrant phospho-Rab signalling and disrupted mitochondria–lysosome communication. (**C**) Activated LRRK2 promotes ESCRT-dependent membrane repair while suppressing lysosomal biogenesis through inhibition of MiT/TFE transcriptional pathways. (**D**) Cellular outcomes are determined by the dynamic balance between LRRK2 kinase activity and phosphatases such as PPM1H, which regulate Rab phosphorylation in space and time. (**E**) LRRK2 functions as a stress-responsive signalling hub integrating immune responses, lysosomal integrity, organelle quality control, and Rab-dependent trafficking. (**F**) Persistent phospho-Rab accumulation disrupts ciliogenesis, axonal autophagosome transport, and organelle homeostasis. (**G**) Genetic risk factors associated with PD, including *GBA1*, *VPS35* (D620N), *ATP13A2*, and *TMEM175*, converge on lysosomal dysfunction and phospho-Rab signalling pathways. (**H**) Proposed unifying model in which lysosomal, mitochondrial, and inflammatory stress signals drive membrane-associated LRRK2 activation, sustained Rab phosphorylation, trafficking defects, chronic cellular dysfunction, and ultimately Parkinson’s disease progression. Created in BioRender. Ledesma Aparicio, J. (2026) https://BioRender.com/wcffkux.

Collectively, these findings reposition LRRK2 as both a sensor and effector of lysosomal stress rather than a constitutively active cytosolic kinase. Lysosomal damage—whether driven by infection, genetic mutations or age-related decline—emerges as a unifying upstream signal that activates LRRK2 and rewires Rab-dependent trafficking. This framework provides a mechanistic bridge between diverse genetic risk factors in PD and highlights lysosomal integrity as a central determinant of LRRK2-driven pathology. From a translational perspective, it suggests that effective therapeutic strategies may need to normalize not only kinase activity but also the cellular contexts—particularly lysosomal health—in which LRRK2 is engaged. The mechanisms linking lysosomal stress and membrane damage to LRRK2 activation are summarized in Table 8 and integrated into the broader cellular model shown in Figure 2.

## 10. LRRK 1, 3, and 4: Evolutionary Divergence, Structure, and Function

In addition to LRRK2, mammals express a closely related paralogue, LRRK1, which shares the canonical ROCO domain architecture yet diverges markedly in regulation, structure, and physiological function. LRRK1 is slightly smaller than LRRK2 and notably lacks the N-terminal ARM domain that underpins Rab-mediated membrane recruitment and feed-forward activation of LRRK2. In cells, LRRK1 localizes predominantly to endosomal compartments rather than to lysosomes or the cytosol. In humans, loss-of-function frameshift mutations that abolish *LRRK1* kinase activity cause autosomal recessive osteosclerotic metaphyseal dysplasia, a severe skeletal disorder characterized by impaired bone remodelling and osteoporosis, underscoring an essential role for LRRK1 in bone homeostasis rather than in neurodegeneration [104]. To date, no convincing genetic or clinical evidence links *LRRK1* to PD.

Despite these divergent disease associations, LRRK1 and LRRK2 share a conserved biochemical function as Rab-directed kinases. LRRK1 selectively phosphorylates Rab7A at Ser72, a Switch II residue equivalent to the sites targeted by LRRK2 in its Rab substrates [65,105]. This specificity is striking: LRRK1 does not phosphorylate canonical LRRK2 substrates such as Rab8A or Rab10, and conversely, LRRK2 does not target Rab7A. Mutations in LRRK1 that are structurally analogous to pathogenic LRRK2 variants—including K746G and I1412T, corresponding to LRRK2 R1441G and I2020T, respectively—as well as Y971F, predicted to destabilize the inactive conformation, markedly enhance Rab7A phosphorylation. These parallels indicate that conserved allosteric mechanisms govern activation across LRRK family members, even as their substrate repertoires have diverged.

Recent work has begun to illuminate the downstream consequences of LRRK1-mediated phosphorylation of Rab7A. Phosphorylated Rab7A selectively binds Pacer, a positive regulator of autophagy, through an RH-domain–dependent interface that recognizes the modified Switch II motif. This finding links LRRK1 activity directly to autophagic regulation and suggests that additional phospho-Rab7A–specific effector proteins remain to be identified. Although the Rab-directed phosphatase PPM1H can dephosphorylate Rab7A in overexpression systems, definitive evidence that endogenous PPM1H serves as the physiological Rab7A phosphatase is currently lacking, implying that distinct phosphatase networks may have evolved for different LRRK–Rab signalling axes [17,105,106].

High-resolution structural studies have provided important insight into the molecular distinctions between LRRK1 and LRRK2. Cryo–electron microscopy structures of kinase-inactive C-terminal RCKW fragments, full-length monomeric LRRK1, truncated variants, and dimeric assemblies reveal several defining features. Unlike LRRK2, LRRK1 lacks the hinge helix within the leucine-rich repeat domain and instead contains an extended C-terminal α-helix. Moreover, LRRK1 lacks the exposed surface patches within the ROC domain that mediate LRRK2’s microtubule binding. In monomeric LRRK1, the LRR domain does not sterically occlude the kinase active site, rendering the catalytic domain accessible to substrates. By contrast, in dimeric assemblies, substrate access is blocked in trans by the ANK domain, which shields the kinase domain of the opposing protomer. This arrangement is conceptually analogous to the autoinhibitory role of the LRR domain in monomeric LRRK2. Consistent with this architecture, mutations that favour monomerization modestly enhance LRRK1 activity, supporting the view that the monomer is the enzyme’s active signalling state [21,107,108].

Regulation of LRRK1 also diverges sharply from that of LRRK2 at the level of upstream signalling. LRRK1 is directly activated by phosphorylation mediated by members of the protein kinase C (PKC) family. PKC phosphorylates a cluster of conserved residues—Ser1064, Ser1074, and Thr1075—within an extended COR-B loop that has no counterpart in LRRK2. Structural modelling has yielded two non-mutually exclusive mechanisms for this activation. One model proposes that the COR-B loop stabilizes the DYG-out inactive conformation by packing against the kinase domain and that phosphorylation or mutation of a critical linchpin residue (Phe1065) relieves this constraint. An alternative model suggests that phosphorylation promotes interaction between the COR-B loop and the αC-helix, stabilizing the catalytically competent conformation. Definitive resolution of these mechanisms will require structural snapshots of PKC-phosphorylated LRRK1 captured in defined conformational states [107,108,109].

From an evolutionary perspective, LRRK family members exemplify how a conserved domain scaffold can be repurposed for distinct biological functions. Metazoan LRRK1 proteins share a WD40 loop that contributes to dimerization and an extended COR-B loop conserved across vertebrates, echinoderms, and spiralians. Intriguingly, the PKC phosphorylation sites and the linchpin Phe1065 residue are restricted to jawed vertebrates, paralleling the emergence of exposed ROC-domain surfaces in LRRK2 that mediate microtubule binding. These patterns indicate lineage-specific adaptations that underlie the distinct regulation, substrate specificity, and disease associations of LRRK1 and LRRK2 [107,110].

Phylogenetic analyses suggest that an early metazoan gene duplication generated four LRRK families. Subsequent lineage-specific losses resulted in vertebrates retaining *LRRK1* and *LRRK2*; arthropods and nematodes retaining only *LRRK3*; spiralians and echinoderms retaining *LRRK1–3*; and cnidarians preserving all four paralogues. Comparative structural modelling highlights substantial divergence among these proteins. LRRK2 is unique in possessing an N-terminal ARM domain and hinge helix. Drosophila LRRK3 contains an expanded ankyrin domain with a large insertion predicted to form a globular β-sheet structure, along with a distinct α-helical region corresponding to the LRRK2 hinge helix but appears to lack the conserved C-terminal α-helix found in LRRK1 and LRRK2. In contrast, cnidarian LRRK4 harbours a large insertion within the COR domain predicted to be intrinsically disordered [107,110].

These differences carry important implications for experimental models. Widely used organisms such as *Caenorhabditis elegans* and *Drosophila melanogaster* express only LRRK3, which differs substantially from mammalian LRRK1 and LRRK2 in domain composition and regulatory logic. Consequently, mechanistic insights derived from these systems must be interpreted cautiously when extrapolating to human LRRK2 biology and PD pathogenesis [6,107,110].

A conserved biochemical feature nonetheless unites LRRK1, LRRK2, and LRRK3: all contain an atypical DYG motif rather than the canonical DFG motif at the magnesium-binding site of the kinase domain. This position corresponds to the glycine mutated in the common LRRK2 G2019S variant. By contrast, LRRK4 retains the canonical DFG motif, as do ancestral non-metazoan LRRK homologues, such as those from *Dictyostelium discoideum*. This pattern suggests that LRRK1–3 evolved from an ancestral LRRK4-like kinase, followed by divergence in catalytic regulation [107,110]. Together, these comparative insights underscore that, despite a shared evolutionary origin, LRRK family members have undergone profound specialization in regulation, function, and disease relevance. The principal structural, regulatory, functional and evolutionary differences among LRRK family members are summarized in Table 9.

## 11. Reframing LRRK2 in Parkinson’s Disease: A Unifying Perspective

The accumulated evidence across structural biology, cell signalling, and human genetics converges on a central conclusion: LRRK2 is not adequately explained as a hyperactive kinase driving PD, but rather as a dynamically regulated signalling system whose dysfunction reflects a failure of coordination across molecular states, cellular compartments, and biological contexts. This reframing emerges not from a single line of inquiry, but from the integration of multiple, previously disconnected observations.

Genetic studies first positioned *LRRK2* as a major contributor to PD [1,2], with pathogenic variants converging on increased kinase activity [29,30]. However, this interpretation becomes insufficient when considered alongside structural and biochemical data. Cryo–electron microscopy and integrative modelling demonstrate that LRRK2 exists as a conformational ensemble, in which long-range interdomain communication, oligomerization, and membrane recruitment collectively determine functional output [11,12,13,21]. Mutations across distinct domains—ROC, COR, kinase, and even N-terminal scaffolding regions—converge by biasing this ensemble toward active-like states, rather than simply increasing catalytic efficiency [24]. Crucially, pharmacological inhibition does not bypass this principle: type I inhibitors stabilize closed conformations, whereas type II inhibitors favour inactive states, producing divergent cellular effects despite similar suppression of kinase activity [6,12]. These findings indicate that conformational state, not kinase activity per se, is the dominant variable controlling LRRK2 biology.

A second axis of convergence lies in the identification of Rab GTPases as the principal substrates of LRRK2 [14,15]. This discovery provided a mechanistic bridge between kinase activity and cellular function, linking LRRK2 to vesicular trafficking, ciliogenesis, and autophagy. Nevertheless, the quantitative features of this system challenge conventional signalling paradigms. Even under conditions of pathogenic activation, only a small fraction of Rab proteins is phosphorylated, and yet this is sufficient to produce substantial cellular effects. This apparent discrepancy is resolved by considering the spatial organization of signalling: phosphorylated Rabs act as local molecular switches, recruiting specific effector proteins such as RILPL1 and reorganizing membrane domains [33,39]. The identification of PPM1H as a Rab-specific phosphatase further establishes that LRRK2 operates within a kinase–phosphatase circuit, in which rapid turnover and compartmentalised regulation define signalling output [17]. Thus, LRRK2 signalling is best understood as a spatiotemporally restricted and dynamically balanced process, rather than a bulk phosphorylation cascade.

A third, and perhaps most transformative, insight is the recognition that LRRK2 is tightly coupled to lysosomal biology. Lysosomal stress—induced by chemical perturbation, genetic defects, or infection—drives recruitment and activation of LRRK2 at membrane surfaces [18,19]. This places LRRK2 within a broader cellular response to organelle damage, linking it to pathways governed by other PD-associated genes, including *GBA1*, *ATP13A2* and *TMEM175* [5,20]. In this context, LRRK2 acts as both a sensor and an effector of membrane integrity, coordinating Rab phosphorylation, effector recruitment, and organelle remodelling [33]. Importantly, this framework provides a mechanistic explanation for the heterogeneity of PD pathology and for the incomplete penetrance of *LRRK2* mutations [37,38]: disease risk reflects not only genetic variation, but also the capacity of cells to maintain lysosomal and trafficking homeostasis over time.

Integration of immune biology further extends this model. LRRK2 is highly expressed in myeloid cells and genetically linked to inflammatory diseases such as Crohn’s disease and leprosy [6]. Experimental evidence shows that pathogen-induced membrane damage activates LRRK2-dependent Rab signalling in macrophages [19], indicating that LRRK2 participates in host defence pathways. This raises a critical possibility: PD may, in part, represent a late-life consequence of dysregulated stress-response signalling that is evolutionarily conserved across tissues. In this view, neuronal degeneration reflects context-dependent vulnerability within a shared immune–lysosomal network, rather than a neuron-specific defect.

Taken together, these lines of evidence support a unifying hypothesis:

Parkinson’s disease associated with *LRRK2* arises from maladaptive stabilization of specific conformational and spatial states of a membrane-responsive signalling system, leading to persistent misregulation of Rab-dependent trafficking and organelle homeostasis.

This hypothesis integrates structural dynamics (conformational bias), biochemical output (Rab phosphorylation), cellular context (membrane recruitment and lysosomal stress), and organismal biology (immune–neuronal interactions). It also explains several otherwise disconnected observations, including low-stoichiometry signalling with high functional impact, mutation-specific penetrance, and the divergent effects of pharmacological inhibitors.

From a translational perspective, this reframing has direct implications. Strategies that focus exclusively on inhibiting kinase activity may fail to restore physiological regulation and may even introduce new imbalances by trapping LRRK2 in non-native conformations. A more effective approach will likely require modulation of the regulatory system itself, including control of conformational equilibria, spatial restriction of activation, and rebalancing of kinase–phosphatase dynamics. Such interventions would aim not to silence LRRK2, but to restore its capacity to respond appropriately to cellular stress.

**Figure 3:**
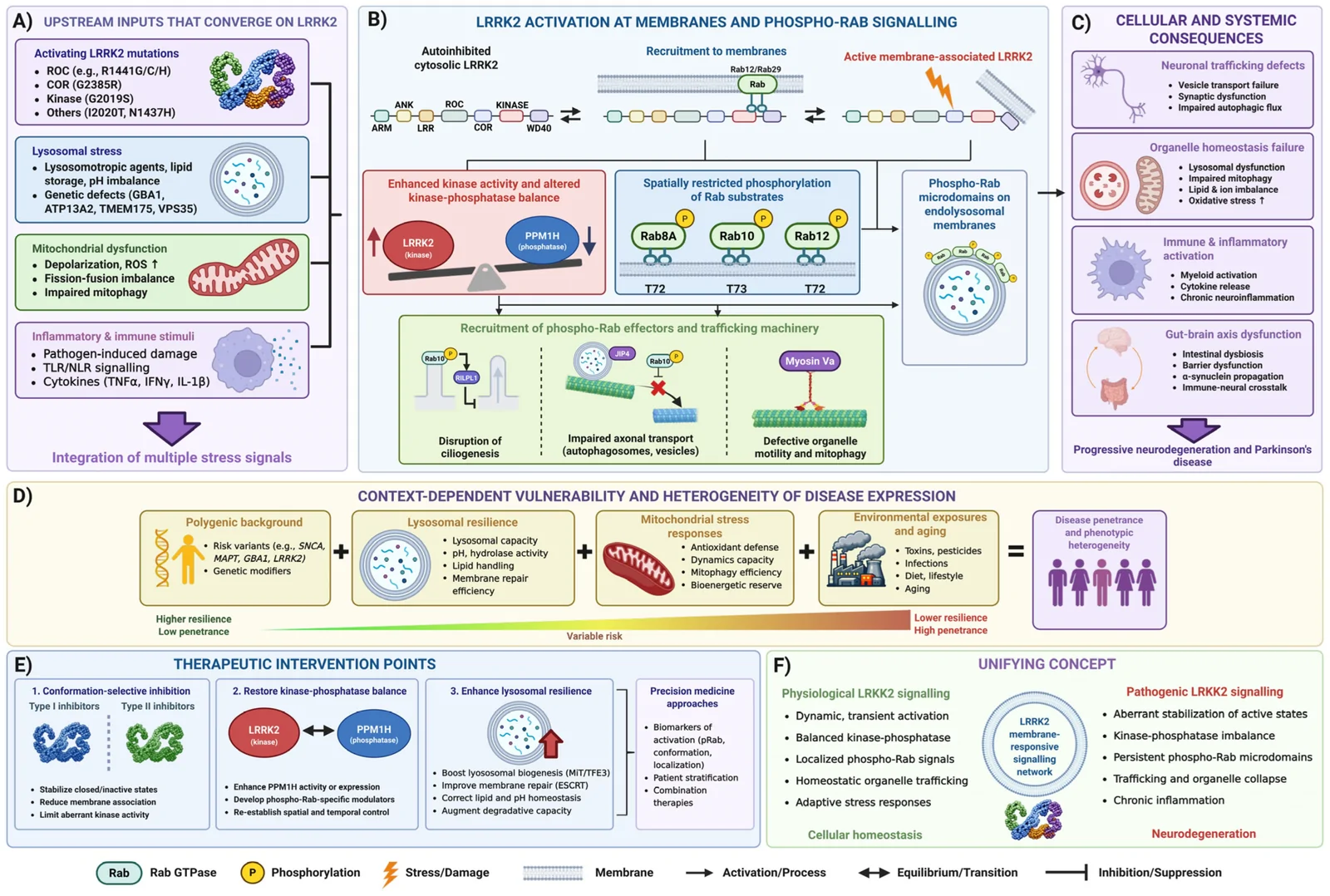
LRRK2 activation integrates lysosomal, mitochondrial, and inflammatory stress signals to regulate Rab-dependent trafficking pathways. (**A**) Multiple upstream factors, including pathogenic *LRRK2* mutations, lysosomal dysfunction, mitochondrial stress, and immune activation, converge on LRRK2 signalling. (**B**) Membrane recruitment of LRRK2, particularly through Rab12- and Rab29-dependent mechanisms, promotes kinase activation and phosphorylation of Rab substrates such as Rab8A, Rab10, and Rab12. Accumulation of phospho-Rabs on endolysosomal membranes recruits trafficking effectors and alters cellular processes including ciliogenesis, axonal transport, organelle motility, and mitophagy. (**C**) Persistent dysregulation of these pathways contributes to neuronal trafficking defects, lysosomal and mitochondrial dysfunction, chronic inflammation, gut–brain axis alterations, and progressive neurodegeneration. (**D**) The clinical expression of disease is influenced by genetic background, lysosomal resilience, mitochondrial adaptive capacity, environmental exposures, and aging, resulting in variable disease penetrance and phenotypic heterogeneity. (**E**) Potential therapeutic strategies include conformation-selective LRRK2 inhibitors, restoration of kinase–phosphatase balance, enhancement of lysosomal function, and precision medicine approaches guided by biomarkers of LRRK2 activity. (**F**) Proposed model in which physiological LRRK2 signalling supports organelle homeostasis and adaptive stress responses, whereas persistent activation drives phospho-Rab accumulation, trafficking defects, organelle failure, chronic inflammation, and neurodegeneration. Created in BioRender. Ledesma Aparicio, J. (2026) https://BioRender.com/bjwy5r1.

In summary, these findings support a systems-level model in which LRRK2 functions as a stress-responsive coordinator of membrane dynamics, trafficking fidelity, organelle homeostasis, and immune signalling. Progressive dysregulation of this interconnected network may undermine cellular organization and promote Parkinson’s disease pathogenesis, as illustrated in Figure 3.

## 12. Discussion

The maturation of the LRRK2 field has brought it to a decisive inflection point. What initially appeared to be a relatively linear narrative—pathogenic kinase hyperactivation causing PD—has evolved into a far more intricate and conceptually demanding framework [3]. The central challenge confronting the field today is no longer data generation but the integration of structural dynamics, membrane biology, lysosomal stress, immunity, and human genetics into a unified, predictive model of disease [5,6].

At the heart of this challenge lies a fundamental reframing of LRRK2 itself. LRRK2 cannot be adequately described as a kinase that toggles between inactive and active states. Instead, convergent structural, biochemical, and cellular evidence reveal a protein that functions as a conformationally plastic, membrane-responsive signalling system [6,12,13]. Kinase activity is necessary but not sufficient to explain LRRK2 biology. Pathogenic mutations, Rab engagement, lysosomal damage, immune stimuli, and pharmacological inhibitors all reshape the conformational landscape of LRRK2 in distinct ways, stabilizing states that may not normally be populated under physiological conditions [12,13]. This exposes a critical vulnerability in prevailing therapeutic strategies: by focusing narrowly on catalytic inhibition, we risk enforcing nonphysiological conformations that disrupt native regulatory equilibria. A major intellectual task for the coming decade will therefore be to move beyond static structural snapshots toward dynamic, time-resolved models that capture how LRRK2 transitions between functional states in living cells.

A closely related conceptual challenge concerns the nature of Rab phosphorylation itself. LRRK2 modifies only a minute fraction of its Rab substrates, yet these low-stoichiometry events exert disproportionately large biological effects [14]. This behaviour defies classical kinase-signalling paradigms predicated on bulk phosphorylation and demands a shift toward models of spatial precision and signal amplification. Phosphorylated Rabs act as local molecular switches, recruiting specialized effector proteins that reprogramme vesicular trafficking, ciliogenesis, and organelle positioning [33]. Understanding LRRK2 signalling, therefore, requires not only identifying substrates but mapping where and when Rab phosphorylation occurs, how long these signals persist, and how they are erased. The discovery of the Rab-directed phosphatase PPM1H was a watershed moment, yet it simultaneously underscored the extent to which our understanding remains incomplete [17]. The identity, compartmentalization, and regulation of additional phospho-Rab phosphatases—and how kinase–phosphatase balance is tuned across cell types—represent a major unresolved frontier.

Perhaps the most disruptive insight to emerge in recent years is the repositioning of lysosomes from passive downstream casualties to active upstream regulators of LRRK2. Lysosomal damage, infection, and trafficking defects are now recognized as potent triggers of LRRK2 recruitment and activation [18,19]. This reframes PD as a disorder deeply rooted in organelle stress management rather than solely in neuronal protein aggregation [5,93]. LRRK2 appears to be engaged at sites of membrane damage, where it coordinates Rab phosphorylation, effector recruitment, and organelle remodelling [33]. Whether this response is initially adaptive—promoting repair and survival—or intrinsically pathogenic remains unresolved. A particularly pressing question is temporal: neurons may be vulnerable not because LRRK2 is neuron-specific, but because they endure chronic, low-grade lysosomal stress accumulated over decades. Dissecting how acute, reversible LRRK2 activation transitions into chronic, maladaptive signalling in ageing brains represents one of the most important challenges ahead [97].

Equally transformative is the recognition that immune biology is not peripheral to LRRK2 function but central to it. Genetic associations with Crohn’s disease, leprosy, and tuberculosis, together with high LRRK2 expression in myeloid cells, reveal a protein positioned at the intersection of host defence and cellular homeostasis [6,19]. The apparent paradox that modest kinase activation predisposes to inflammatory disease, while other activating mutations drive neurodegeneration, exposes the inadequacy of one-dimensional models of pathogenicity. Instead, disease risk appears to emerge from the interaction between mutation-specific effects and tissue-specific signalling environments [3]. This immune–lysosomal–neuronal axis offers a powerful framework for reinterpreting PD not as an isolated neurodegenerative disorder, but as a late-life manifestation of broader dysregulation in stress-response and defence pathways.

These insights also force a critical reassessment of experimental models. Many widely used organisms do not express LRRK2, but instead encode structurally divergent paralogues governed by distinct regulatory logic [6]. Although such systems have yielded valuable mechanistic clues, their limitations are now evident. Future progress will depend on human-relevant models that preserve the unique structural features, regulatory interactions, and cellular contexts of LRRK2. Patient-derived neurons and glia, immune–neural co-culture systems, organoids with intact lysosomal and immune signalling, and integrative human genetics will be essential to bridge the gap between molecular insight and clinical relevance.

All of these challenges converge on a profound therapeutic question: should LRRK2 be silenced, or should its activity be rebalanced? Evidence from human genetics indicates that heterozygous loss-of-function variants in *LRRK2* are well tolerated, yet complete suppression may not be benign, given LRRK2’s roles beyond the nervous system [75]. The future of LRRK2-targeted therapy likely lies in strategies that restore physiological regulation rather than abolish activity—biasing conformational equilibria, modulating membrane recruitment, recalibrating Rab phosphorylation dynamics, or selectively intervening under pathological contexts such as chronic lysosomal stress [6,12,13]. Achieving this will require biomarkers that report not only kinase activity, but also conformation, localization, and network state.

In sum, the LRRK2 field has entered a phase in which incremental advances are no longer sufficient. What is required is a conceptual synthesis that integrates structure, dynamics, cell biology, immunity, and human disease. If successful, this effort will not merely refine our understanding of LRRK2 but will also reshape how Parkinson’s disease is conceptualized—as a disorder of dynamic cellular decision-making rather than a static proteinopathy [5]. Such a shift would represent not just progress, but a genuine change in the intellectual architecture of neurodegeneration research.

## Figures and Tables

**Table 1:** Structural organization and conformational regulation of LRRK2.

Structural Layer	Key Elements	Mechanistic Insight	Functional Consequence	References
Global architecture	RCKW (ROC–COR–kinase–WD40); J-shaped fold	Domain alignment → physical coupling of ROC–COR ↔ kinase	Enables GTPase–kinase crosstalk → integrated signalling output	[11]
Full-length organization	ANK/LRR surrounding kinase region; extended ARM domain	ANK–LRR/core contacts → stabilization of open/autoinhibited architecture	Restricts kinase accessibility → low basal activity	[21]
Domain dynamics	Mobile armadillo repeats; constrained LRR	Mobility ↔ rigidity balance → coupling localization to catalysis	Partner binding → modulation of kinase output	[21,23]
Kinase conformations	Closed (active-like) vs. open (inactive DYG-out) states	Inhibitor class → conformational selection (type I vs. II)	Distinct cellular phenotypes despite similar inhibition	[12,22]
Allosteric coupling	Helical bridges linking ROC–COR ↔ kinase N-lobe	Interdomain tethering → long-range allostery	Constrains basal state → permits rapid activation switching	[11,23]
Catalytic core	Bilobal kinase domain; misaligned inactive state	Structural misalignment → inefficient catalysis	Explains low basal activity → autoinhibited ensemble	[12,21]
Disease mutations	PD-linked variants (e.g., activating substitutions)	Disrupted interdomain communication → shift of the conformational ensemble toward kinase-competent states	↑ kinase activity; ↓ biomarker phosphorylation (Ser935/955/973)	[12,14,24]
Microtubule assemblies	Helical filaments (e.g., I2020T mutant)	Oligomerization on MTs → higher-order packing interfaces	Context-dependent MT binding → unclear physiological role	[25,26,27]
Membrane recruitment	Rab29-dependent tetrameric complex	Spatial assembly → active central protomers + inactive periphery	Enables compartmentalized activation at membranes	[13]
Systems-level model	Conformational ensemble + quaternary structure	Recruitment + oligomerization → reshape structural landscape	Activity emerges as ensemble property → pharmacological targeting via state selection	[6,13,22]

Abbreviations: ANK, ankyrin; ARM, armadillo; COR, C-terminal of ROC; DYG, Asp–Tyr–Gly motif; GTPase, guanosine triphosphatase; LRR, leucine-rich repeat; LRRK2, leucine-rich repeat kinase 2; MT, microtubule; PD, Parkinson’s disease; RCKW, ROC–COR–kinase–WD40; ROC, Ras of complex proteins; WD40, WD40 repeat domain. Symbols: →, leads to or results in; ↔, bidirectional interaction or functional coupling; ↑, increased; ↓, decreased.

**Table 2:** LRRK2 signalling, genetics and pathogenic networks in Parkinson’s disease.

Layer	Key Components	Mechanistic Insight	Pathobiological Consequence	References
Genetic basis	*LRRK2* variants (common monogenic PD cause)	Mutations → kinase-dependent pathogenic entry point	Extends beyond familial PD → informs sporadic disease mechanisms	[1,2,3,14]
Protein architecture	Multidomain ROCO protein (LRR–ROC–COR–kinase–WD40)	Modular domains → integrate GTPase + kinase + scaffolding functions	Coordinates trafficking, cytoskeleton, and membrane signalling	[6,11,21]
Mutation hotspots	G2019S (kinase); R1441 (ROC)	Distinct biochemical defects → converge on ↑ kinase activity	Unified outcome → hyperactive/dysregulated signalling state	[14,15,29,30,31]
Rab signalling axis	Rab8, Rab10, Rab12 phosphorylation	LRRK2 hyperactivity → excessive Rab phosphorylation	Disrupted vesicular trafficking → endolysosomal + autophagic dysfunction	[14,15,18,34]
Cellular pathways	Vesicle transport; lysosome; autophagy; synaptic vesicles	Rab-dependent dysregulation → impaired intracellular logistics	Dopaminergic neuron vulnerability → network-level dysfunction	[18,32,33,34,39]
Genetic epidemiology	~10–15% PD genetic; ~3% *LRRK2* carriers	Population-specific allele frequency → ancestry-dependent risk	Highlights stratified genetics → precision medicine need	[3,4,40]
Clinical phenotype	*LRRK2*-PD vs. idiopathic PD	Similar core features → subtle divergence in progression	↓ motor progression; ↓ cognitive burden (subset)	[35,36,41]
Neuropathology	Variable α-synuclein deposition	LRRK2 dysfunction ≠ single proteinopathy	Heterogeneous pathology → multiple disease trajectories	[2,42]
Penetrance & modifiers	30–70% penetrance; polygenic + environmental modifiers	Genetic + environmental interactions → variable expressivity	Challenges deterministic models → complexity in counselling	[37,38,43]
Systems-level model	Kinase-driven pathogenic networks	LRRK2 → integrates trafficking, lysosomal, and immune pathways	Convergent network dysfunction → PD pathogenesis	[5,6,18,19]
Translational framework	Biomarkers + kinase inhibitors + stratification	Genetic stratification → pathway-targeted intervention	Precision therapeutics → extends beyond mutation carriers	[6,40,44,45,46]

Abbreviations: COR, C-terminal of ROC; GTPase, guanosine triphosphatase; LRR, leucine-rich repeat; LRRK2, leucine-rich repeat kinase 2; PD, Parkinson’s disease; Rab, Ras-related protein in brain; ROC, Ras of complex proteins; ROCO, protein family defined by tandem ROC and COR domains; WD40, WD40 repeat domain. Symbols: →, leads to or results in; ↑, increased; ↓, decreased; ≠, not equivalent to; ~, approximately.

**Table 3:** LRRK2 conformational regulation and kinase activation in Parkinson’s disease.

Axis	Elements	Mechanism	Outcome	References
Molecular framework	Multidomain LRRK2 (~288 kDa)	Integrated scaffold + ROC GTPase + kinase → signalling convergence	Central hub → trafficking, cytoskeleton, membrane regulation	[6,11,21]
Domain architecture	ARM–ANK–LRR–ROC–COR–kinase–WD40	Linear organization → long-range interdomain coupling	Activity governed by conformational communication (≠linear enzyme)	[11,21,23]
N-terminal module	ARM/ANK + LRR repeats	Solenoid scaffold → membrane association + intramolecular constraint	Spatial control → modulation of catalytic accessibility	[21,54]
ROCO core	ROC + COR-A/COR-B	GTP binding/hydrolysis → bidirectional ROC ↔ kinase coupling	Nucleotide state → tunes kinase activation	[11,23,31]
Catalytic assembly	Kinase domain + WD40 + αC-helix	Interdomain packing → stabilized conformational states	Maintains regulated active/inactive equilibria	[11,21,23]
Pathogenic principle	Variants across domains	Distributed mutations → bias conformational equilibrium	Convergent phenotype → ↑ kinase activity (shared mechanism)	[14,15,24]
Kinase activation (direct)	G2019S (DYG motif)	Activation loop perturbation → ↑ catalytic efficiency (~2×)	Sustained signalling gain → downstream pathway disruption	[29,30]
Allosteric activation	ROC (R1441G/C/H); COR-B (Y1699C)	↓ GTP hydrolysis/interface destabilization → active-state bias	Strong ↑ kinase output (3–4× via Rab10 phosphorylation)	[14,15,31,55]
Variant distribution	>780 missense; ~50 activating	Non-catalytic mutations → long-range allostery → kinase activation	Confirms system-level regulation (≠active site–centric)	[6,24]
Population risk spectrum	R1628P (COR-A)	Mild conformational shift → modest kinase increase	Partial risk allele → incomplete penetrance continuum	[56,57]
Alternative activation routes	I2020T, T2031S	Structural perturbation (αC-helix/activation segment) → active conformation	Multiple structural paths → same biochemical endpoint	[12,24,52]
Regulatory phosphorylation	Ser910/935/955/973 (CK1α)	Phosphorylation → 14-3-3 binding → cytosolic stabilisation	Maintains inactive state → prevents membrane activation	[49,50,58]
Biomarker logic	“Biomarker sites” (Ser910–973)	Dephosphorylation → kinase activation + membrane recruitment	Inverse activity readout → clinical pharmacodynamic marker	[24,49,59,60]
Autophosphorylation	Ser1292	Direct kinase-dependent phosphorylation → intrinsic activity marker	High specificity but low detectability → translational limitation	[44,53]
Systems model	Conformational ensemble	Interdomain signalling → dynamic state transitions	Pathogenicity = failure of conformational restraint → sustained hyperactivity	[6,13,21,23]
Therapeutic implication	Kinase pathway targeting	Modulate conformational landscape → restore balance	Enables precision therapy beyond mutation-specific strategies	[6,12,22]

Abbreviations: ANK, ankyrin; ARM, armadillo; CK1α, casein kinase 1 alpha; COR, C-terminal of ROC; DYG, Asp–Tyr–Gly motif; GTP, guanosine triphosphate; GTPase, guanosine triphosphatase; kDa, kilodalton; LRR, leucine-rich repeat; LRRK2, leucine-rich repeat kinase 2; Rab, Ras-related protein in brain; ROC, Ras of complex proteins; ROCO, protein family defined by tandem ROC and COR domains; Ser, serine; WD40, WD40 repeat domain. Symbols: →, leads to or results in; ↔, bidirectional interaction or functional coupling; ↑, increased; ↓, decreased; ≠, not equivalent to; ~, approximately; >, greater than; ×, fold change.

**Table 4:** LRRK2–Rab signalling axis: substrate specificity and pathogenic rewiring in Parkinson’s disease.

Axis	Elements	Mechanism	Outcome	References
Substrate discovery	Phosphoproteomics + genetic/chemical orthogonality	LRRK2 (G2019S) ± inhibitors + A2016T control → substrate filtering	Rab10 + pSer935 identified → validated physiological substrates	[14]
Substrate hierarchy	Rab GTPases (dominant); Ser1292 (auto-site)	Convergent validation → Rab phosphorylation as core output	Establishes a unified signalling framework	[14,15,53]
Mutation convergence	Pathogenic *LRRK2* variants	↑ kinase activity → ↑ Rab phosphorylation	Shared biochemical endpoint across mutations	[14,15,24]
Rab repertoire	Rab3, Rab8A/B, Rab10, Rab12, Rab29, Rab35, Rab43	Selective substrate recognition → restricted Rab subset	Targeted rewiring of trafficking pathways	[14,64]
Context amplification	*VPS35* (D620N) background	Retromer dysfunction → ↑ LRRK2 activity → expanded Rab targeting (e.g., Rab1)	Genetic interaction → pathway amplification	[62]
Rab biology	Small GTPases (GDP ↔ GTP cycle)	Nucleotide cycling → vesicle budding, transport, fusion	Maintains membrane identity + trafficking fidelity	[61]
Phosphorylation site	Switch II residues (e.g., Rab10 Thr73, Rab8 Thr72)	LRRK2 phosphorylation → conformational disruption of effector interfaces	Functional uncoupling from regulatory machinery	[14,64]
GDI/GEF disruption	GDIs; Rabin8 (Rab8 GEF)	Phospho-Rab → ↓ GDI binding + ↓ GEF interaction	Rab trapping on membranes → impaired recycling	[14,64]
Effector rewiring	RILPL1/2, JIP3/4, Myosin Va	Phospho-Rab → gain of RH2-mediated binding	Dominant signalling complexes → altered trafficking outputs	[33,64,65]
Stoichiometry principle	<1% Rab phosphorylated	Low-level modification → high-affinity effector recruitment	Signal amplification without global trafficking collapse	[14,15]
Ciliogenesis defect	Rab10–RILPL1 axis	Phospho-Rab10 binding → inhibition of primary cilium formation	Loss of ciliogenesis → impaired Hedgehog signalling	[39,63]
Circuit vulnerability	Striatal cholinergic interneurons	↓ Hedgehog signalling → ↓ GDNF support to dopaminergic neurons	Selective neuronal vulnerability in PD	[39,63]
Axonal/autophagic defects	Autophagosome transport; centrosome cohesion	↑ phospho-Rab → trafficking + structural disruption	Impaired neuronal maintenance + polarity	[34,66]
Biomarker strategy	Phospho-Rab detection (Ab/MS)	Quantification of phospho-Rabs → pathway activity readout	Robust translational biomarkers	[15,44]
Systems model	LRRK2–Rab signalling network	Kinase hyperactivity → selective Rab rewiring	Links genetics → trafficking defects → circuit dysfunction	[6,14,34,39,64]

Abbreviations: Ab, antibody; GDI, GDP dissociation inhibitor; GDP, guanosine diphosphate; GDNF, glial cell-derived neurotrophic factor; GEF, guanine nucleotide exchange factor; GTP, guanosine triphosphate; GTPase, guanosine triphosphatase; JIP3/4, c-Jun N-terminal kinase-interacting proteins 3 and 4; LRRK2, leucine-rich repeat kinase 2; MS, mass spectrometry; PD, Parkinson’s disease; pSer935, phosphorylated serine 935; Rab, Ras-related protein in brain; RH2, RILP homology 2; RILPL1/2, Rab-interacting lysosomal protein-like 1 and 2; Ser, serine; Thr, threonine; VPS35, vacuolar protein sorting-associated protein 35. Symbols: →, leads to or results in; ↔, cycling between states; ↑, increased; ↓, decreased; ±, with or without; <, less than.

**Table 5:** LRRK2 membrane recruitment and Rab-dependent activation dynamics.

Axis	Elements	Mechanism	Outcome	References
Basal distribution	Cytosolic LRRK2 (~90–95%); minor membrane pool	Cytosol ↔ membrane equilibrium → spatial constraint of activity	Membrane localization → ↑ kinase activity	[6,67]
Spatial activation principle	Membrane-bound LRRK2	Rab-mediated recruitment → local activation at membrane surfaces	Spatial confinement → efficient substrate phosphorylation	[18,54,67]
Rab-dependent recruitment	GTP-bound prenylated Rabs	Rab–LRRK2 binding → membrane tethering + catalytic activation	Couples localization → enzymatic output	[54,68]
Rab29 paradigm	Rab29 (PARK16 locus)	Golgi localisation → recruits/activates LRRK2 (overexpression)	Proof-of-principle activator → not universal regulator	[13,68]
Physiological specificity	Rab29 vs. Rab8A/Rab10 abundance	Low Rab29 expression → limited global impact	Cell-type specificity → strong role in immune cells	[54,69]
Cell-type regulation	Macrophages (Rab29); monocytes (Rab38)	Lineage-specific Rab expression → selective activation pathways	Context-dependent LRRK2 activation	[6,71]
Rab-binding Site 1	ARM domain (~residues 360–450)	Binds non-phospho Rabs (Rab29/8A/10) → initial membrane docking	Recruitment of LRRK2 → substrate proximity	[54,68]
Site 1 disruption	L403 mutation	Loss of Rab binding → impaired membrane recruitment	Confirms structural requirement for activation	[54]
Rab-binding Site 2	N-terminal region (Lys17/Lys18)	Selective binding to phospho-Rabs → retention mechanism	Stabilises membrane association → ↑ kinase dwell time	[54]
Feed-forward loop	Site 1 + Site 2 cooperation	Initial Rab binding → phosphorylation → phospho-Rab rebinding	Positive feedback → local kinase amplification	[54]
Membrane microdomains	Phospho-Rab–enriched regions	Cooperative binding → reduced dissociation	High local kinase concentration → effector recruitment	[54]
Rab-binding Site 3	ARM domain (Rab12-specific)	Rab12 interaction → additional activation route	Enhances robustness → context-dependent signalling	[70]
Multisite integration	Sites 1 + 2 + 3	Simultaneous Rab engagement → cooperative binding	Increases activation fidelity + signalling strength	[54,70]
Substrate positioning constraint	Rab Switch II binding	ARM binding → occludes phosphorylation site	Implies separate catalytic docking interface	[54,70]
Catalytic interface (inferred)	Kinase-proximal docking site	Additional binding surface → proper substrate orientation	Enables phosphorylation of Switch II residues	[6,70]
Activation model	Rab-driven cooperative recruitment	Membrane localization → feed-forward phosphorylation loop	LRRK2 = membrane-responsive signalling hub	[13,54,70]
Pathogenic implication	Spatial dysregulation	Aberrant recruitment/amplification → sustained activation	Links localization → disease mechanisms	[6,14,54,70]
Therapeutic insight	Target membrane recruitment	Disrupt Rab–LRRK2 interactions → modulate activation	Alternative to ATP-competitive inhibition	[6,22,54,70]

Abbreviations: ARM, armadillo; ATP, adenosine triphosphate; GTP, guanosine triphosphate; LRRK2, leucine-rich repeat kinase 2; Lys, lysine; PARK16, Parkinson disease susceptibility locus 16; Rab, Ras-related protein in brain; vs., versus. Symbols: →, leads to or results in; ↔, bidirectional equilibrium or translocation; ↑, increased; ~, approximately; =, functions as or is conceptually equivalent to.

**Table 6:** LRRK2 conformational pharmacology: type I versus type II kinase inhibition.

Axis	Type I Inhibitors	Type II Inhibitors	Mechanistic Implication	References
Binding mode	ATP-competitive; closed (active-like) state	Allosteric; inactive (DYG-out) state	Conformation selection → distinct signalling outputs	[12,22,76]
Biomarker sites (Ser910/935)	Dephosphorylation	Preserved phosphorylation	Conformation → controls 14-3-3 binding	[49,50,60,76]
14-3-3 interaction	Lost	Maintained	Structural stability ↔ cytosolic retention	[49,76]
Rab phosphorylation	↓ Rab10 phosphorylation	↓ Rab10 phosphorylation	Shared efficacy despite distinct conformations	[12,14,76]
Cellular effects	↑ aggregation; microtubule association (overexpression)	No MT association; preserved stability	Active-like trapping → structural perturbation	[25,49,59,76]
Safety profile	Lung/kidney alterations (preclinical)	Not yet defined	Toxicity may reflect conformational state (≠inhibition alone)	[16,74]
Development status	Clinically advanced (DNL201, DNL151, MLi-2)	Preclinical (e.g., GZD-824)	Need for conformation-aware optimization	[6,22,45,77,78]

Abbreviations: ATP, adenosine triphosphate; DYG, Asp–Tyr–Gly motif; LRRK2, leucine-rich repeat kinase 2; MT, microtubule; Rab, Ras-related protein in brain; Ser, serine. Symbols: →, leads to or results in; ↔, bidirectional relationship or functional coupling; ↑, increased; ↓, decreased; ≠, not equivalent to.

**Table 7:** PPM1H counter-regulation of LRRK2–Rab signalling.

Axis	Key Elements	Mechanism	Outcome	References
Kinetic separation	Rab10 vs. Ser910/935 dephosphorylation	Rapid Rab dephosphorylation → independent phosphatase control	Defines the fast-responsive arm of the LRRK2 pathway	[14,17]
Phosphatase identity	PPM1H	RNAi screen → selective Rab10 dephosphorylation	Direct counterbalance to LRRK2 kinase activity	[17]
Substrate specificity	Rab8A/B, Rab10 (≠Rab12 in cells)	Selective binding → phospho-Rab recognition	High-fidelity substrate discrimination	[17,79]
Structural determinant	PPM1H “flap” domain	Unique structural insert → confers Rab specificity	Molecular basis of selective dephosphorylation	[80]
Spatial regulation	Golgi-localised PPM1H	Subcellular localization → substrate accessibility	Explains Rab selectivity in cells	[79]
Parallel phosphatases	Residual Rab dephosphorylation	PPM1H loss → incomplete effect	Indicates additional phosphatase activities	[17,79]
Functional leverage	PPM1H modulation	↓ PPM1H → ↑ phospho-Rab without altering LRRK2	Enables pathway dissection independent of the kinase	[17]
Cellular outcomes	Ciliogenesis; autophagosome transport	Phospho-Rab accumulation → impaired trafficking processes	Links Rab signalling → neuronal dysfunction	[17,34]
Systems model	Kinase–phosphatase balance	LRRK2 ↔ PPM1H → dynamic phosphorylation equilibrium	Defines tunable signalling circuit (≠linear cascade)	[17,34,79,80]

Abbreviations: LRRK2, leucine-rich repeat kinase 2; PPM1H, protein phosphatase magnesium/manganese-dependent 1H; Rab, Ras-related protein in brain; RNAi, RNA interference; Ser, serine; vs., versus. Symbols: →, leads to or results in; ↔, dynamic balance or functional coupling; ↑, increased; ↓, decreased; ≠, not equivalent to.

**Table 8:** Lysosomal stress–driven activation of LRRK2 signalling.

Axis	Key Elements	Mechanism	Outcome	References
Lysosomal activation	Chloroquine; lysosomal swelling	Lysosomal stress → LRRK2 recruitment to membranes	Local activation → Rab phosphorylation (e.g., Rab8A)	[18]
Infection-triggered activation	Mycobacterium, Listeria, Candida	Phagosomal damage → LRRK2 recruitment → Rab8A phosphorylation	ESCRT recruitment → membrane repair coordination	[19]
Membrane damage signalling	LLOME; lysosomal rupture	Membrane breach → LRRK2 activation at lysosomes	Stress-responsive kinase behaviour (≠basal cytosolic)	[19,81]
STING–CASM signalling	STING; CASM; GABARAP	STING activation → CASM-dependent GABARAP lipidation → LRRK2 recruitment	Lysosomal LRRK2 activation → Rab phosphorylation	[82]
Lysosomal repair context	ESCRT; Ca^2+^–sphingomyelin; ER contacts	Damage → rapid repair pathways activation	Provides a platform for LRRK2 recruitment and signalling	[83,84,85,101]
Pathway integration in PD	*LRRK2*, *GBA1*, *ATP13A2*, *TMEM175*	Lysosomal dysfunction → convergent PD pathway	Central role of the lysosome in disease pathogenesis	[3,6,89]
Downstream trafficking	JIP4; lysosomal tubulation	LRRK2 activation → phospho-Rab recruitment → JIP4 binding	Lysosome remodelling + altered positioning	[33]
Transcriptional control	TFE3	Active LRRK2 → ↓ TFE3 nuclear localisation	↓ lysosomal biogenesis + proteolytic capacity	[93,94]
Genetic interaction	*GBA1* variants	↓ glucocerebrosidase activity → lipid accumulation	Links lipid dysregulation → lysosomal stress → LRRK2 activation	[95,96,97]
Recruitment mechanism	Rab12; phospho-Rab effectors	Rab mislocalization → LRRK2 recruitment (partial redundancy)	Feed-forward Rab phosphorylation loop	[54,70,102]
Feed-forward amplification	RILPL1; TMEM55B; RUFY3	Phospho-Rabs → effector binding → membrane retention	Sustained LRRK2 activation + trafficking defects	[54,103]
Genetic amplifier	*VPS35* (D620N)	Retromer dysfunction → lysosomal stress → ↑ LRRK2 activity	Strong Rab phosphorylation + PD phenotype	[62,100,103]
Systems model	Lysosomal stress axis	Damage → recruitment → Rab phosphorylation → retention	LRRK2 = sensor + effector of lysosomal integrity	[6,18,19,33,94,102]

Abbreviations: ATP13A2, ATPase type 13A2; Ca^2+^, calcium ion; CASM, conjugation of ATG8 to single membranes; ER, endoplasmic reticulum; ESCRT, endosomal sorting complexes required for transport; GABARAP, GABA type A receptor-associated protein; GBA1, glucosylceramidase beta 1; JIP4, c-Jun N-terminal kinase-interacting protein 4; LLOME, L-leucyl-L-leucine methyl ester; LRRK2, leucine-rich repeat kinase 2; PD, Parkinson’s disease; Rab, Ras-related protein in brain; RILPL1, Rab-interacting lysosomal protein-like 1; RUFY3, RUN and FYVE domain-containing protein 3; STING, stimulator of interferon genes; TFE3, transcription factor E3; TMEM55B, transmembrane protein 55B; TMEM175, transmembrane protein 175; VPS35, vacuolar protein sorting-associated protein 35. Symbols: →, leads to or results in; ↑, increased; ↓, decreased; ≠, not equivalent to; =, functions as or is conceptually equivalent to.

**Table 9:** LRRK family divergence: structure, regulation and function.

Axis	LRRK1	LRRK2	Other LRRKs (3/4)	Mechanistic Implication	References
Domain architecture	Lacks ARM domain	Contains ARM + hinge helix	Divergent insertions (ANK, COR, disordered regions)	Structural divergence → distinct recruitment and regulation modes	[21,107,108]
Subcellular localisation	Endosomal	Cytosolic ↔ membrane (Rab-dependent)	Variable across species	Compartmentalisation → pathway specificity	[54,67,105]
Substrate specificity	Rab7A (Ser72)	Rab8A, Rab10, etc.	Conserved Rab targeting (family-wide)	Divergent Rab selectivity → specialised trafficking roles	[14,64,105,106]
Activation mechanisms	PKC-dependent phosphorylation (COR-B loop)	Rab-mediated recruitment + allostery	Distinct lineage-specific regulation	Upstream control divergence → signalling diversity	[13,54,109]
Allosteric regulation	Monomer–dimer equilibrium → activity control	Conformational ensemble → kinase regulation	Conserved DYG motif (LRRK1–3)	Shared catalytic logic → distinct regulatory tuning	[21,23,107]
Downstream function	Autophagy (Pacer via phospho-Rab7A)	Vesicular trafficking, lysosome, cytoskeleton	Context-dependent	Functional specialisation → pathway diversification	[14,33,105,111,112]
Disease association	Bone disorder (osteosclerotic dysplasia)	Parkinson’s disease	Limited/unclear	Divergence → tissue-specific pathology	[1,2,104]
Evolutionary trajectory	Vertebrate retention	Vertebrate retention	LRRK3 (invertebrates); LRRK4 (ancestral-like)	Gene duplication → lineage-specific loss and adaptation	[107,110]
Model system limitation	Not present in common models	Not conserved in invertebrates	LRRK3 only (e.g., Drosophila, *C. elegans*)	Translational gap → caution in extrapolation	[6,107,110]

Abbreviations: ANK, ankyrin; ARM, armadillo; *C. elegans*, *Caenorhabditis elegans*; COR, C-terminal of Ras of complex proteins; DYG, Asp–Tyr–Gly motif; LRRK, leucine-rich repeat kinase; Pacer, protein associated with UVRAG as autophagy enhancer; PKC, protein kinase C; Rab, Ras-related protein in brain; Ser, serine. Symbols: →, leads to or results in; ↔, bidirectional equilibrium or functional coupling.

## Data Availability

No new data were created or analyzed in this study. Data sharing is not applicable to this article. All graphs presented in the figures are schematic or representative illustrations and were not generated from original experimental or clinical data.

## Abbreviations

The following abbreviations are used in this manuscript:

ANK	Ankyrin repeat domain
ARM	Armadillo repeat domain
ATP	Adenosine triphosphate
ATP13A2	ATPase cation transporting 13A2
COR	C-terminal of ROC
cryo-EM	Cryo–electron microscopy
DYG	Aspartate–tyrosine–glycine motif
ESCRT	Endosomal sorting complexes required for transport
GBA1	Glucosylceramidase beta 1
GDI	GDP dissociation inhibitor
GDP	Guanosine diphosphate
GTP	Guanosine triphosphate
GTPase	Guanosine triphosphatase
JIP4	c-Jun N-terminal kinase-interacting protein 4
LLOME	L-leucyl-L-leucine methyl ester
LRRK	Leucine-rich repeat kinase
PD	Parkinson’s disease
PKC	Protein kinase C
PPM1H	Protein phosphatase, Mg^2+^/Mn^2+^ dependent 1H
Rab	Ras-related protein in brain
RCKW	ROC–COR–kinase–WD40 catalytic region
RILPL1	Rab-interacting lysosomal protein-like 1
ROC	Ras of complex proteins
ROCO	ROC–COR-containing protein family
TMEM175	Transmembrane protein 175
VPS35	VPS35 retromer complex component
WD40	WD40 repeat domain
